# Shear-Responsive Supramolecular Preformed Particle Gel: Tailoring Network Architectures for Selective Water Blocking

**DOI:** 10.3390/polym18070850

**Published:** 2026-03-31

**Authors:** Simon López-Ramírez, Víctor Matías-Pérez, José F. Barragán-Aroche, Luis E. Díaz-Paulino, Raúl Oviedo-Roa, Oscar González-Antonio, Elba Xochitiotzi-Flores

**Affiliations:** 1 Facultad de Química/USIP, Universidad Nacional Autónoma de México, Ciudad Universitaria, Ciudad de México 04510, Mexicoledp@quimica.unam.mx (L.E.D.-P.); 2 Faculty of Earth Sciences, Universidad Autónoma de Nuevo León, Linares 67700, Mexico; 3 Instituto Mexicano del Petróleo, Eje Central Lázaro Cárdenas Norte 152, Del. Gustavo A. Madero, Ciudad de México 07730, Mexico; 4 Departamento de Química Orgánica, Facultad de Química, Universidad Nacional Autónoma de México, Ciudad Universitaria, Ciudad de México 04510, Mexico

**Keywords:** polyampholyte, double network, hybrid, preformed particle gels, water control, water shutoff, shear thickening, PPG, hydrogel

## Abstract

Managing excessive water production in oil fields during primary, secondary, or enhanced recovery remains challenging. It increases costs and reduces hydrocarbon recovery, particularly in reservoirs with high-conductivity pathways such as high-permeability zones and fractures. Hydrogels are commonly used for water blocking and retention; however, their effectiveness diminishes at higher flow rates due to mechanical weaknesses and structural limitations. These problems are intensified under harsh environmental conditions, including high temperatures, salinity, and hardness. In this study, we investigate how altering the molecular suprastructure of preformed particle gel (PPG) can improve its effectiveness in shear-responsive water-blockage treatments, particularly when traditional PPGs cannot control rising flow rates. We enhance the shear-responsive mechanical properties of a composite PPG by increasing the density and diversity of intermolecular interactions. We use two different strategies: first, incorporating cationic groups into the polymer backbone to form a polyampholyte network with stronger electrostatic interactions; second, adding a linear anionic polymer to generate a secondary interpenetrating network that can undergo a coil–stretch transition under thermal and shear stimuli, thereby enhancing its own solvation and whole-network expansion. Molecular simulations provide an interpretation of the experimentally observed shear-thickening response and enhanced disproportionate permeability reduction at high flow rates. The water residual resistance factor of the improved PPGs deviates from the typical shear-thinning power-law behavior (*n* < 1) observed in conventional PPG, showing shear-thickening (*n* > 1). Tests reveal a strong ability to preferentially reduce water flow over oil, with Disproportionate Permeability Reduction increasing from 8 to 117 in the high-flow-rate zone. The enhanced strength and thermal stability also improve resistance to washout under high-pressure gradients. This research provides a novel approach to tailoring the microscopic architecture of PPGs to achieve selective, robust water blockage, offering a high-efficiency solution for complex reservoir environments.

## 1. Introduction

Preformed particle gel (PPG) technology offers an alternative to in situ gel for water shutoff and conformance control. PPG consists of polymer network particles produced in industrial settings, ensuring consistent quality and performance. This technology prevents unpredictable, harmful polymerization reactions within the oil reservoir, unlike in situ gels. In above-ground oil field facilities, PPG is swollen with production water and injected into the oil reservoir. Once inside, properly sized and property-matched PPG particles preferentially enter high-permeability channels, avoiding low-permeability zones within the porous formation. The selective placement significantly reduces the likelihood of formation damage [1,2,3,4].

Fluid mobility and capillary forces govern the flow of water and oil into oil reservoirs. Water is generally less viscous; in the absence of capillary forces, it flows more readily through high-conductivity channels or fractures [5]. Such conditions frequently result in water breakthroughs, in which water reaches the producer wells before the oil, thereby trapping most of the oil in the reservoir. PPG provides a technological solution to address this reservoir anomaly. Once placed in the medium, PPG acts as a selective barrier, reducing the system’s overall permeability. However, oil’s permeability remains higher than water’s, leading to disproportionate permeability reduction (DPR) or relative permeability modification [6]. Water is retained in the PPG-packed media due to strong electrostatic, dipole–dipole, and hydrogen-bond interactions with PPG molecules, creating a highly water-wet surface. Conversely, weak van der Waals forces prevent oil from adhering to the hydrophilic PPG surface. This high repellency allows oil to pass through while water remains trapped [7].

The residual resistance factor (RRF) measures the effectiveness of water-blocking treatments by comparing fluid mobility before and after PPG injection. It quantifies the reduction in permeability in porous media, serving as a key indicator of treatment success. A DPR occurs when the RRF for water exceeds that for oil, indicating more effective water blockage. Experimental studies have shown that RRF decreases with increasing flow rate, following a power-law relationship [3,6,8,9,10,11,12], highlighting the shear sensitivity of PPG performance.

This behavior indicates that the plugging strength of PPGs is shear-sensitive: lower flow rates allow gel particles to accumulate, pack, and deform efficiently, yielding stronger, more stable gel packs with better sealing and higher RRF, whereas higher flow rates may cause damage, degradation, or erosion, leading to washouts from the medium and resulting in lower RRF and DPR [12]. The interpretation suggests that as flow increases, it exerts stress on the PPG, creating new pathways in the media or enlarging existing spaces where flow can increase, thereby reducing the blocking ability in fractures, high-conductivity channels [6], or porous media located around producer wells [11]. The balance between covalent crosslinks and physical interactions within the swollen network intrinsically governs PPG functional performance. Under increasing shear conditions, however, conventional PPG systems exhibit reduced mechanical stability, leading to diminished structural integrity and flow-modulating capacity.

Typically, PPGs exhibit mechanical weaknesses, characterized by low fracture energy and reduced elastic modulus [13,14]. To address this issue, an improved design can modify PPGs’ mechanical properties to achieve high strength and fracture toughness while maintaining sufficient stiffness to withstand loads, especially given their low density of crosslinked molecules in water. Enhancing mechanical properties through molecular structural changes involves manipulating molecular characteristics at a fundamental level. This includes increasing the density of physical crosslinking using supramolecular interactions, such as hydrogen bonds or ionic interactions [15]. Achieving these properties with fully covalently crosslinked hydrogels is challenging because the polymer network remains permanently bound by strong, irreversible covalent bonds, which can lead to polymer degradation [16]. Modifying simple hydrogel frameworks can enable the creation of multifunctional engineered gels, such as polyampholyte and hybrid gels.

A polyampholyte hydrogel is a network with positive and negative charges randomly distributed throughout. The conformation of the resulting polymer chain and the network structure improve mechanical properties, enabling the final matrix to better resist temperature fluctuations, salinity, and shear stress. Notably, this type of hydrogel can swell and remain stable even at high ionic strengths [17,18,19,20,21,22,23,24]. A hybrid hydrogel consists of two interpenetrating networks. The first is a cross-linked polymer structure that provides a scaffold for the hydrogel, giving it elasticity and flexibility. The second is typically a soft, tough, and loosely cross-linked polymer framework. These two networks are interconnected through physical or chemical crosslinking. When external forces are applied, the two networks work together to absorb and dissipate stress, thereby enhancing the hydrogel’s toughness and crush resistance [24,25,26,27].

In this study, we enhance the mechanical behavior of a composite deformable preformed particle gel (C-PPG) [28,29] by engineering its supramolecular architecture [30], yielding two gel types: polyampholyte and hybrid. These enhanced molecular suprastructures were designed to improve water-blocking by inducing DPR in water-oil systems, particularly at high water flow rates, where traditional PPGs become less effective. The polyampholyte PPG (P-PPG) was synthesized by incorporating a cationic monomer into the C-PPG backbone, while the hybrid (H-PPG) was created by embedding an anionic polymer within the C-PPG. We also performed a computational analysis of the suprastructural motifs in these PPGs to elucidate the supramolecular mechanisms governing structure–property relationships in these reinforced PPG systems.

The new P-PPG was synthesized by incorporating an optimized concentration of the cationic monomer diallyldimethylammonium chloride (DADMAC). Because this monomer lacks acid-base activity, resistance to syneresis remains unchanged. In addition to hydrogen bonding and hydrophobic interactions, coulombic attraction between oppositely charged monomers drives phase transitions in the original C-PPG structure. The new H-PPG was created by integrating an optimized concentration of an anionic, partially hydrolyzed polyacrylamide (HPAAm) into the C-PPG framework. In H-PPG, networks interact via pendant groups from both the added polymer and the main network, as well as with inorganic ions in water. This leads to the formation of a supramolecular complex structure. Previous research indicates that flexible, diverse non-covalent cross-linking plays a vital role in regulating swelling behavior within the PPG matrix. Improved mechanical properties arise from covalent bonds, hydrogen bonds, electrostatic interactions, and the relaxation of dispersed polymer chains [30].

The C-PPG platform used in this work [28,29] was previously developed and validated for structural integrity under high-temperature and high-salinity conditions, demonstrating stable swelling behavior and resistance to syneresis. Building on this established network architecture, the present study advances the system through deliberate supramolecular engineering. Rather than modifying macroscopic operational parameters, we focus on molecular-level reinforcement by increasing the density and diversity of non-covalent interactions within the polymer matrix. This approach enables a systematic investigation of how controlled architectural modifications influence mechanical stability and shear-responsive behavior in hydrated polymer networks. The structure’s monomers address specific challenges: sodium 2-acrylamido-2-methylpropane sulfonate (AMPSNa) provides resistance to heat and salinity by hindering hydrolysis of nearby amide groups through steric effects and electrostatic repulsion; vinylpyrrolidone (VP) enhances the chemical stability of acrylamide-based polymers under high-temperature and high-salinity conditions; acrylamide (AAm) shapes the gel’s structure and, together with clay nanoparticles, influences its mechanical properties. N,N-methylenebis(acrylamide) (MBA) forms covalent bonds that create the backbone of the gel network.

## 2. Materials and Methods

Materials were obtained from Aldrich (St. Louis, MO, USA) and used without further purification. These included acrylamide (AAm, 99%), vinylpyrrolidone (VP, 98%), 2-acrylamido-2-methylpropane sulfonic acid (AMPSa, 99%), and diallyldimethylammonium chloride (DADMAC, 65%) monomers; N, N-methylenebis(acrylamide) (MBA, 99%) served as a crosslinker; ammonium persulfate (APS, 99%) was used as a free-radical initiator; and N, N, N′, N′-tetramethylethylenediamine (TEMED, 99%) was the catalyst. Gargon Industrial provided bentonite (BE). SNF supplied 40% hydrolyzed polyacrylamide (HPAAm, MW 10^7^ Da). Nitrogen with (99.995%) was purchased from Praxair. The synthesis of the sodium salt of AMPS (AMPSNa) has been described elsewhere [31]. n-Decane (95%) was obtained from Aldrich and used without purification. Purified deionized water (DW) was produced using a Milli-Q Integral 5 system (Millipore, Molsheim, France).

Modified Bentonite (MB) was prepared by placing a flask with a 5 wt.% BE suspension in DW and sonicated for 2 h in an ultrasonic bath without temperature control. The sonication was performed with an Aquasonic 150T (VWR Scientific, Radnor, PA, USA) at 135 W. The production brine (Table 1) is a mixture of produced water from different oil reservoir layers. Its composition matches typical oil-field brines in southeastern Mexico and is readily available at oil-field sites for PPG swelling.

### 2.1. Synthesis and Characterization of Preformed Particle Gels

The P-PPG and H-PPG compositions used in this study were optimized to ensure structural stability and viscoelastic properties under extreme reservoir conditions (130 °C, 84,000 mg/L salinity, and 4000 mg/L hardness). The concentration ranges of the components evaluated during optimization are documented in Tables S12 and S13 of the Supporting Information.

#### 2.1.1. Composite PPG (C-PPG)

**Figure 1:**
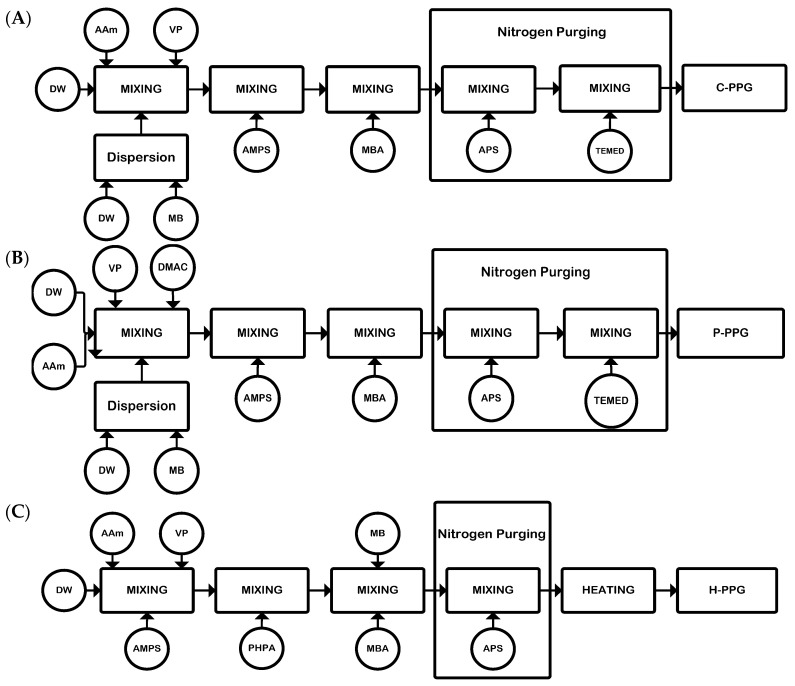
Synthesis of preformed particle gels. (**A**) Composite (C-PPG), (**B**) Polyampholyte (P-PPG), and (**C**) Hybrid (H-PPG).

The C-PPG was synthesized with a total monomer concentration of 30 wt.% and a 1:1:1 molar ratio of AAm, VP, and AMPSNa [28]. To prepare 150 g, AM and VP were added separately to a 250 mL beaker equipped with a magnetic stirrer, and the mixture was stirred vigorously until uniform. Then, DW and 60 g of MB were added, followed by AMPSNa. The gelant solution was stirred continuously until all monomers were fully dissolved. Next, 0.75 g of MBA crosslinker was added while stirring continued. A nitrogen purge was initiated and maintained for 0.5 h. Next, 0.15 g of APS initiator and 0.075 g of TEMED catalyst were added, and the nitrogen purge was completed. Exothermic polymerization began almost immediately, resulting in hydrogel formation within 0.5 h. A diagram of the C-PPG synthesis process is shown in Figure 1A, and all component details are summarized in Table 2.

#### 2.1.2. Polyampholyte PPG (P-PPG)

The P-PPG was synthesized with a total monomer concentration of 30 wt.% and a molar ratio of 4:1:1:1 for AAm, VP, AMPSNa, and DADMAC, respectively. A total of 150 g of P-PPG was produced as follows: in a 250 mL beaker with a magnetic stirrer, 60 g of MB was mixed with DW and stirred vigorously for 1 h. After this initial mixing, the monomers AAm, VP, DADMAC, and AMPSNa were gradually added to the reaction mixture, with stirring maintained for 0.16 h between additions. Subsequently, 0.375 g of MBA crosslinker was added. To purge oxygen, nitrogen was bubbled through the mixture for 30 min. Finally, 0.075 g of APS initiator and 0.045 g of TEMED catalyst were added to the reaction, with TEMED added slowly. The polymerization occurred at room temperature, and hydrogel formation was observed within 0.5 h. A brief outline of P-PPG synthesis is shown in Figure 1B, with all component details summarized in Table 2.

#### 2.1.3. Hybrid PPG (H-PPG)

The H-PPG was synthesized at a total monomer concentration of 30 wt.% with a 1:1:1 molar ratio of AAm, VP, and AMPSNa. To prepare 150 g, monomers VP, DW, AAm, and AMPSNa were sequentially added to a 250 mL beaker equipped with a magnetic stirrer, and the mixture was vigorously stirred to ensure thorough blending. Each addition was stirred for 0.16 h before the next was added. Then, 3 g of HPAAm was incorporated and stirred vigorously for 3 h. Subsequently, 0.75 g of MBA and 15 g of MB were added sequentially, and the mixture was stirred for 1 h to ensure complete dispersion. Nitrogen gas was bubbled through the mixture for 0.5 h to remove oxygen. Finally, 0.225 g of APS initiator was added, and the primary network polymerization was performed in an oven at 60 °C for 0.5 h. A schematic overview of the H-PPG synthesis process is shown in Figure 1C, with all component details summarized in Table 2.

#### 2.1.4. Characterization

Infrared spectra were recorded using an Agilent FTIR Cary 600 ATR spectrometer (Agilent, Santa Clara, CA, USA). The solid-state ^13^C-NMR spectra of the compound were obtained using a JEOL 600 MHz spectrometer (JEOL, Tokyo, Japan) operating at 150 MHz, equipped with a 1H broadband decoupler in a 4 mm broadband probe. A spinning rate of 15–18 Hz effectively removed spinning sidebands. An optimized cross-polarization contact time of 0.5 ms was employed. Additionally, dipolar dephasing experiments (^13^C CPMAS NQS) were conducted with a 30 µs delay before activating the 1H decoupler.

The complete characterization included elemental analysis, TGA/DSC, and environmental scanning electron microscopy, as outlined in the Supporting Information.

### 2.2. Swelling, Syneresis Resistance, and Mechanical Stability

The gellant solution was either left in a beaker or poured into a mold shaped like a film (0.08 cm × 0.4 cm × 0.4 cm) to permit polymerization. To produce particles, the gel was collected from the beaker, cut into small pieces, and dried in a vacuum oven at 60 °C for 24 h. The dried PPG was then ground and sifted until particles measuring 106 µm were obtained. The films were used without further processing.

The swelling assessment involves immersing a dry sample of PPG particles in production brine at room temperature for 24 h, until equilibrium is reached. The swollen particles are then carefully removed from the liquid and blotted on filter paper to eliminate excess water. Finally, the weight of the swollen particles is measured.

The equilibrium swelling ratio (ESR) is determined by the following equation [4]:

(1) $$ESR=\;\frac{W_{s}-W_{d}}{W_{d}}$$

where $W_{s}$ and $W_{d}\;$ refer to the masses of the hydrogel when it is swollen and dried, respectively.

The syneresis resistance evaluation starts by weighing a dry PPG sample. Then, immerse the particles in production brine at room temperature for 24 h until swelling reaches equilibrium. Next, gently remove the swollen particles from the liquid and blot them on filter paper to eliminate any residual water. Transfer 8 g of the swollen PPG particles into 25 mL glass tubes filled with production brine. Seal the tubes with Teflon caps and place them in small stainless-steel containers fitted with O-ring screw caps. Age the samples for 90 days at 130 °C. After this aging process, carefully separate the particles from the liquid, blot them on filter paper to remove excess water, and record the final weight. The syneresis factor (SF) is then calculated using the following formula.

(2) $$SF=\left(\frac{W_{s}^{i}-W_{s}^{f}}{W_{s}^{i}}\right)*100$$

where $W_{s}^{i}$ and $W_{s}^{f}$ represent the initial and final weights of the PPG during aging.

To evaluate the mechanical stability during aging, PPG films were swollen in excess production brine and aged at 130 °C in a 250 mL capped beaker. The storage ($G^{\prime }$) and loss ($G^{\prime \prime }$) moduli were measured using a controlled-stress rheometer (MCR-501, Anton Paar) with a 25 mm parallel-plate geometry and a 1 mm gap. Over 90 days, the aged films were removed from the oven weekly, cooled, and cut to 25 mm in diameter to fit the rheometer plate. Using these samples, we identified the linear viscoelastic (LVE) region and performed a semi-quantitative rheological assessment of the hydrogel’s properties. The LVE region was determined within a strain range of 0.01% to 0.3% at a constant angular frequency of 10 rad/s. A deformation of 0.1% was used for viscoelastic analysis across the frequency range of 0.1 to 100 rad/s. The viscoelastic properties of all samples were measured at 25 °C.

### 2.3. Residual Resistance Factor and Disproportionate Permeability Reduction

n-Decane and production brine were employed to assess PPG’s effectiveness as a selective permeability modifier by examining the relationship between differential pressure and flow rates.

**Figure 2:**
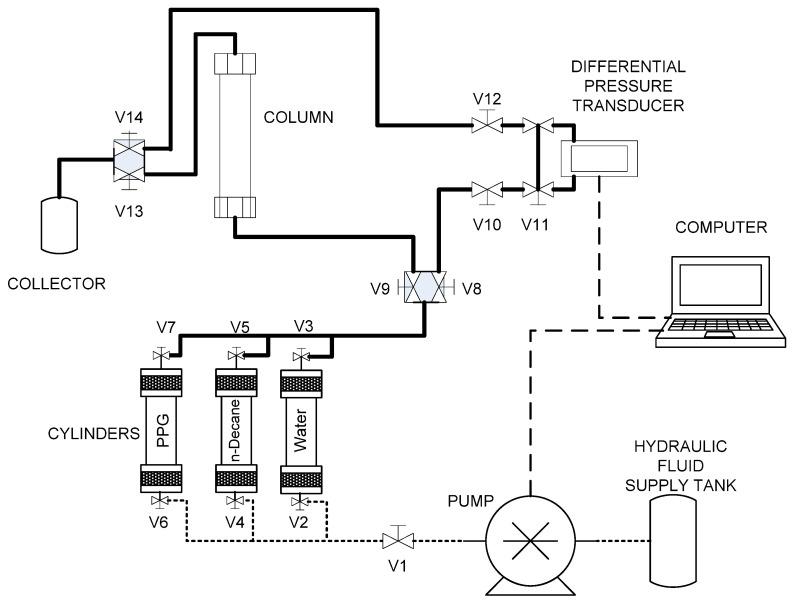
Experimental setup used to evaluate the Disproportionate permeability reduction in preformed particle gels. The setup comprises: (1) an automatic displacement pump (Quizix (Broken Arrow, OK, USA), QX6000); (2) cylinders containing oil, water, or PPGs; (3) a rechargeable thin column with an inner diameter of 0.305 cm and a length of 60.0 cm; (4) a differential pressure gauge (Validyne (Canoga Park, CA, USA), P55); and (5) a cumulative liquid collector. Line definitions: solid (evaluated fluid samples), dashed (pressurization hydraulic fluid), and long-dashed (electronic communication between instruments).

After polymerization, the gel was collected and cut into 1 cm^3^ pieces. The pieces were vacuum-dried at 60 °C for 24 h. Once dried, the PPG was ground and sieved to obtain particles measuring 1.4 mm. These particles were soaked in production brine at room temperature for 24 h. After absorbing water and swelling, the particles were transferred to the PPG cylinder and then injected into the column (Figure 2). A 200-mesh cap was installed at the column outlet to prevent PPG particles from being washed out. Then, water or oil was injected until a steady state was reached, and the pressure difference (Δ*P*) was recorded. The experiment was performed at various flow rates, and the corresponding Δ*P* values were used to calculate the Residual Resistance Factor (RRF). This factor provides a reliable measure of a gel’s ability to generate significant pressure gradients during fluid flow. For a specific fluid, the calculation was performed using the following equation [10,12]:

(3) RRFf=λf,bλf,a=kμf,bkμf,a=kf,bkf,a= q L μfA ∆Pf,bq L μfA ∆Pf,a=∆Pf,a∆Pf,b

where

λ = Fluid mobility.

k = Medium permeability.

$\mu_{f}$ = Fluid viscosity of phase “f”.

q = Volumetric flow rate.

L = Length of porous medium.

A = cross-sectional area perpendicular to flow.

∆P = Pressure Drop across the length L.

The subscripts “*b*” and “*a*” refer to measurements taken before and after PPG placement, respectively, while “*f*” indicates the water or oil phases.

The performance of the PPGs was evaluated by analyzing the RRF’s behavior using the Matías-Pérez deterministic model [12], which explains how the RRF varies with flow rate.

(4) $${RRF}_{f}=\frac{D\;p_{1}}{A\;k_{0}\;p_{1}\pm \mu_{f}\;L\;q}$$

where:

D = Shape-dependent geometry factor for the PPG pack.

The “*+*” and “−” signs indicate channeling and compaction of the PPG, respectively. The parameters for pressure ${\;p}_{1}$ and initial permeability $k_{0}$ are obtained by fitting the experimental data ${\;p}_{1}\;$ represents the stabilized pressure provided by the PPG once fluid channeling occurs. Under this condition, the flow rates increase, ${\;p}_{1}$ remains constant, and the RRF tends to zero. Conversely, when the PPG is compacted, it forms a barrier that restricts fluid movement. In this situation, ${\;p}_{1}\;$ corresponds to the pressure required to establish the barrier. As a result, flow rates decrease, causing the RRF to rise and approach infinity. In all cases, $k_{0}\;$ indicates the initial permeability of the PPG-packed medium.

The effectiveness of DPR occurs when ${RRF}_{w}$ is greater than ${RRF}_{o}$. This effectiveness can be assessed at each flow rate using:

(5) DPR=RRFwRRFo=kw,bkw,ako,bko,a =ko,akw,a= qLμoA∆Po,aqLμwA∆Pw,a=∆Pw,a∆Po,aμoμw

${\left[k\right]}_{w,b}\;$ and ${\left[k\right]}_{o,b}$ refer to the absolute permeability of the empty column, and these values are equal.

The subscripts “*b*” and “*a*” refer to measurements taken before and after PPG placement, respectively, while “*w*” and “*o*” indicate the water and oil phases.

### 2.4. Computational Analysis

The computational analysis treats the H-PPG hydrogel as a hybrid matrix composed of the terpolymer C-PPG and 40% hydrolyzed acrylamide (HPAAm), with both components modeled as two-unit oligomers. Likewise, the P-PPG hydrogel consists of two polymers: PA-PPG, which has an open structure with an allylic pendant group, and PB-PPG, which exists in a cyclic pyrrolidinium form. These polymers were modeled as single-unit oligomers. Oligomer lengths were chosen to produce fragments of similar mass and spatial extension, enabling a fair comparison of their supramolecular architectures. The molecular geometries of all oligomers were optimized using semi-empirical quantum-mechanical methods in Gaussian 09 [32,33].

To examine the stability of unswelled hydrogels, supramolecular complexes made of two optimized oligomers were created using the Blends module of Materials Studio (MS) [34]. This module conducts extensive random sampling of millions of possible relative orientations between rigid oligomers, evaluates their interaction energies with classical force fields, and identifies the lowest-energy configuration within the explored conformational space. The supramolecular complexes include: (i) C-PPG oligomer dimers, modeling the C-PPG hydrogel; (ii) HPAAm oligomer dimers, modeling the HPAAm hydrogel; (iii) C-PPG/HPAAm oligomer pairs, modeling the H-PPG hydrogel, with the former and latter assigned the roles of base and screen in Blends, respectively; (iv) P_A_-PPG oligomer dimers; and (v) P_A_-PPG/P_B_-PPG pairs, where the former acts as base and the latter as screen.

Once the lowest-energy configurations were identified, the geometries of the supramolecular complexes were relaxed using the Forcite module of MS, followed by a comprehensive optimization at the Density Functional Theory (DFT) level. The resulting DFT electron densities were then employed to analyze intermolecular interactions through the molecular electrostatic potential (ESP) and to characterize their topologies within the framework of the Quantum Theory of Atoms in Molecules (QTAIM), as implemented in the Multiwfn program [35]. The ESP describes the energy interaction between the molecular charge distribution and a positive test charge placed at a specific point in space, providing a physically meaningful representation of regions susceptible to electrophilic or nucleophilic interactions.

The thermodynamic feasibility of the swelling process undergone by the polymers in formation water with high cation content was evaluated by calculating the Flory–Huggins interaction parameter ($\chi_{FH}$), as implemented in the MS Blends module. This parameter measures the affinity and structural compatibility between two species and shows how it depends on temperature [36], providing thermodynamic insight into polymer–solvent interactions and how these are affected by cation concentrations in brines. The $\chi_{FH}$ values were determined at both room temperature and 92 °C for interactions between the oligomeric models of C-PPG, H-PPG, HPAAm, P_A_-PPG, and P_B_-PPG with the main formation-water components, specifically H_2_O, Na^+^, Ca^2+^, and Mg^2+^.

The calculated reaction energies are not intended to replicate polymerization kinetics but rather to provide a comparative thermodynamic measure of the relative stabilization of the representative oligomeric motifs used to model the different PPG suprastructures.

## 3. Results and Discussion

### 3.1. Characterization of the H-PPG and P-PPG

**Figure 3:**
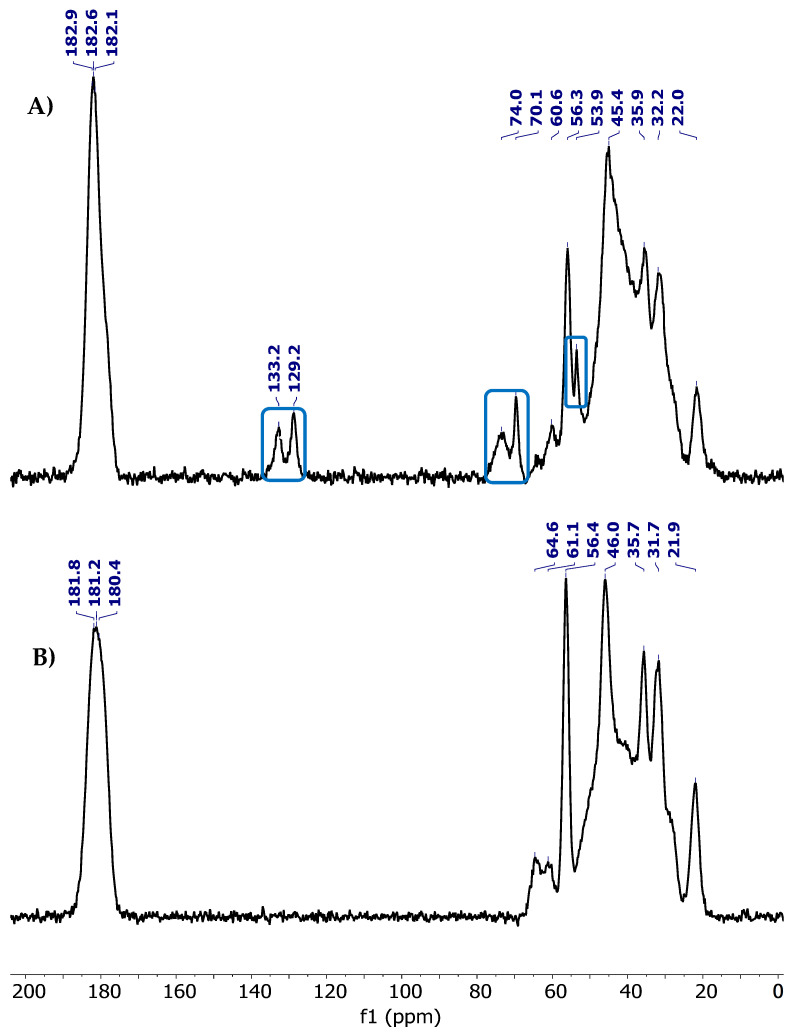
^13^C NMR CP/MAS spectra of PPGs acquired at 150 MHz. (**A**) P-PPG terpolymer synthesized from the monomers AAm, VP, AMPSNa, and DADMAC. (**B**) H-PPG terpolymer synthesized from the monomers AAm, VP, and AMPSNa. Blue frames highlight signals associated with the DADMAC structure.

Figure 3 shows the ^13^C NMR CP-MAS spectra, and Table 3 lists the signals identified as carbon atoms in P-PPG and H-PPG. The spectra display distinctive signals ranging from 21.9 to 64.9 ppm for H-PPG and from 22.0 to 60.6 ppm for P-PPG. These signals are attributed to the aliphatic carbons present in the -CH_3_, -CH_2_-, -CH-, and -C groups derived from the monomers AAm, VP, and AMPSNa used in the synthesis. Additionally, three carbonyl carbon signals (C=O) are observed at 180.4–181.8 ppm for H-PPG, and 182.1–182.9 ppm for P-PPG, confirming the presence of amide and carboxy groups in the material. Also, three signals highlighted in the blue box in Figure 3A, at chemical shifts of 53.9, 70.1, and 74.0 ppm, are associated with DADMAC in P-PPG. In contrast, the signals at 129.3 and 133.2 ppm indicate that a portion (CH=CH-CH-) of the DADMAC structure did not undergo polymerization [37,38]. The integration of monomers into the P-PPG and H-PPG backbones was confirmed by comparing their spectra with those of the homopolymers poly-AAm, poly-VP, and poly-AMPS, as detailed in the Supporting Information (Figures S5–S10, Tables S4 and S5).

**Figure 4:**
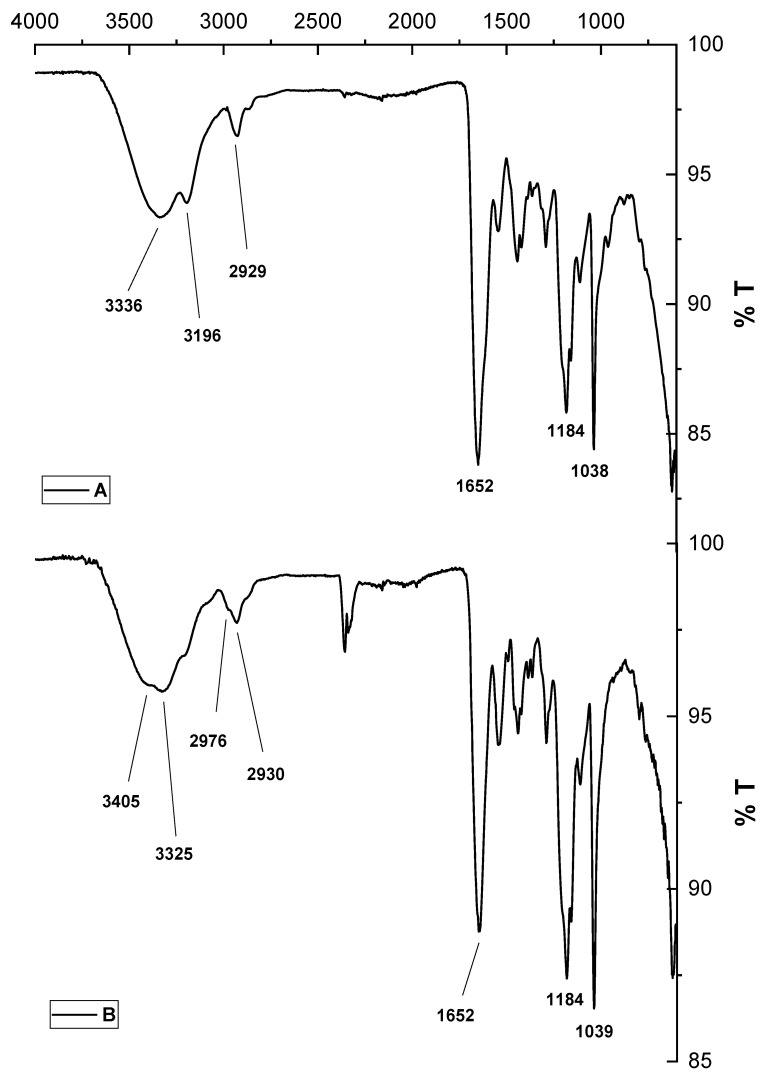
FTIR-ATR spectra of PPGs obtained by attenuated total reflectance. (**A**) P-PPG terpolymer synthesized from the monomers AAm, VP, AMPSNa, and DADMAC. (**B**) H-PPG terpolymer synthesized from the monomers AAm, VP, and AMPSNa.

FTIR-ATR spectroscopy was performed on the terpolymers H-PPG and P-PPG (Figure 4 and Table 4). The identified bands with average wavenumbers included: for H-PPG, resonance was observed at 3405 cm^−1^ and 3325 cm^−1^, and for P-PPG at 3336 cm^−1^, both indicating -NH/-NH_2_ groups; the -CH_2_ and -CH_3_ groups showed bands at 2976 cm^−1^ and 2936 cm^−1^ for H-PPG; P-PPG displayed bands at 3196 cm^−1^ and 2929 cm^−1^; a band at 1652 cm^−1^ appeared in both H-PPG and P-PPG, indicating the -C=O of the amide group; the -C(CH_3_)_2_- group exhibited a band at 1184 cm^−1^ for both polymers; and H-PPG showed a band at 1039 cm^−1^, and P-PPG at 1038 cm^−1^, both related to the -S=O group. Additionally, the spectra of the homopolymers poly-AAm, poly-VP, and poly-AMPS were compared with those of the terpolymers H-PPG and P-PPG. Spectra are presented in Figures S11–S16 and Tables S6 and S7 in the Supporting Information.

Supplementary characterization, detailed in the Supporting Information, confirms that the synthesis produced hydrogels with the intended composition. Elemental analysis (Tables S8–S10) verifies the presence of all expected elements, while TGA/DSC results (Figures S17–S20) show thermal behavior consistent with the constituent homopolymers. Furthermore, the morphology of the porous structures confirms the formation of a polymer network, with each PPG retaining its essential characteristics. The H-PPG double network displays a dense, uniform structure upon swelling in formation water (Figure S22), whereas the P-PPG swollen in deionized water exhibits a narrow, uniform pore-size distribution, which contributes to its enhanced overall mechanical properties (Figure S21).

### 3.2. Swelling, Syneresis Resistance, and Mechanical Stability

Four main factors influence the swelling ability of gels: polymer-solvent interactions, ionic forces, electrostatic forces, and the elastic contractile forces within the polymer network [39]. In swollen PPG, ionic forces are the strongest. These forces originate from Coulombic attraction or repulsion between ions with opposite or similar charges, caused by mobile ions in the brine and from the polar functional groups of the PPG structure. Attraction can also be influenced by electrostatic interactions, such as hydrogen bonding, as well as weaker forces, like dipole–dipole interactions, that occur between polar PPG molecules or between PPG molecules and mobile ions. Interactions between PPG and water arise from the strong attraction between the polar pendant functional groups of the PPG network and water molecules. This creates a significant driving force, called osmotic pressure, which draws in and retains water, leading to swelling that varies with the size and shape of the counterions [40]. Additionally, electrostatic repulsion between similarly charged groups on the polymer backbone causes the polymer chains to disperse, further increasing swelling. Elastic forces generated by the crosslinked PPG network resist excessive expansion and help return the hydrogel to its collapsed state, thus limiting maximum swelling. In addition to these forces, crosslink density is another critical factor that significantly affects overall swelling; lower density permits greater expansion.

Both C-PPG and H-PPG display an equilibrium swelling ratio (ESR) of 11 (Table 5). The H-PPG structure remains stable and behaves similarly to C-PPG at 60 °C because there is not enough energy for HPAAm to switch from a coiled to an extended, linear form. P-PPG exhibits stronger ionic interactions compared to C-PPG and H-PPG, due to the additional attraction between its positively charged groups and brine anions, as well as electrostatic interactions between the anionic and cationic groups in the PPG backbone, resulting in a low ESR value of 4.39.

After aging for 90 days at 130 °C, the low syneresis factor (SF) values for C-PPG and P-PPG (SF ≈ 0.3) indicate high structural stability. This stability results from the incorporation of the functional groups VP, DADMAC, and AMPSNa, which enhances the polymer network’s resistance to hydrolysis, the primary cause of chemical degradation [28]. With aging, the H-PPG exhibited significant weight gain (SF ≈ −53.4), mainly due to increased water absorption, which was influenced by the HPAAm polymer chains. Dispersed HPAAm coils tend to stretch, expand, or deform under thermal stress. This transition from a dense, high-entropy, low-volume structure to a more extended, lower-entropy, higher-volume form enhances interactions with solvent molecules, mobile ions, and the main PPG matrix, thus changing their solvation state [41].

**Figure 5:**
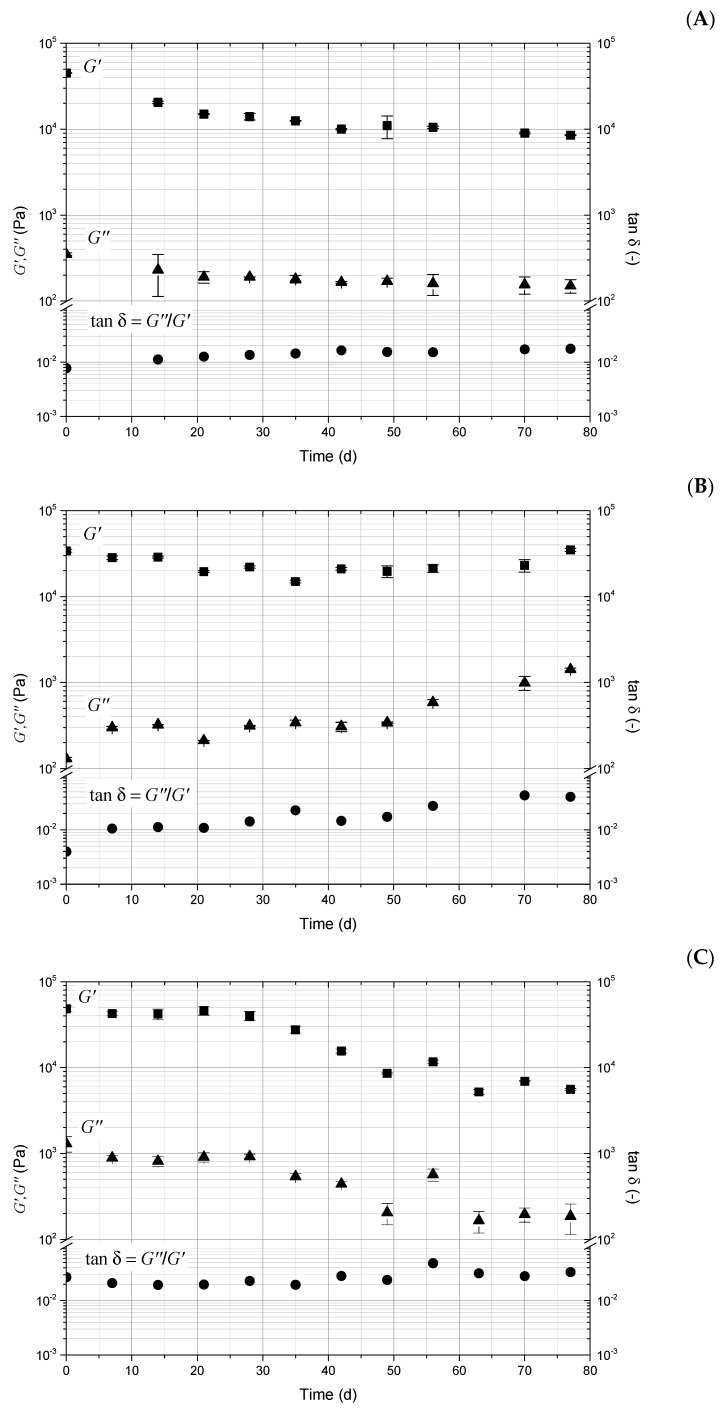
The viscoelastic behavior of PPG samples aged at 130 °C, measured at 10 rad/s and 0.1% strain over time. (**A**) C-PPG. (**B**) P-PPG. (**C**) H-PPG. The mean of three determinations and the standard deviation are shown.

The viscoelastic behavior of PPG samples was examined over a 90-day aging period (Figure 5). Modulus data were obtained from the complete viscoelastic curves at an angular frequency of 10 rad/s. These curves were measured at a constant strain of 0.1% across an angular frequency range from 0.01 to 100 rad/s. The time-dependent evaluation shows that the storage modulus ($G^{\prime }$) surpasses the loss modulus ($G^{\prime \prime }$), indicating a significant elastic contribution to deformation. Furthermore, the loss factor (tanδ=G″G′) remains relatively constant over time. This consistent behavior, along with $G^{\prime }$ > $G^{\prime \prime }$, indicates that the systems exhibit solid-like rather than viscous-liquid behavior throughout the aging process. This demonstrates that the hydrated, three-dimensional, cross-linked network remains structurally stable during this period [16,42,43]. The Supporting Information shows the linear viscoelastic region for all samples analyzed in this section (Figures S23–S25).

The elastic modulus of C-PPG (Figure 5A) decreases with increasing temperature; the first supramolecular interactions that break down or weaken are the weakest non-covalent forces, specifically dipole–dipole interactions. After this, the resulting network remains stable, as indicated by the constant elastic modulus (≈10,000 Pa). In contrast, P-PPG maintains its elastic strength (≈20,000 Pa) as temperature increases (Figure 5B) and remains stable throughout aging. The enhanced stability results from stronger ionic interactions between cationic and anionic charges, originating from the DADMAC monomer and brine ions. Initially, H-PPG (≈45,000 Pa) exhibits a higher elastic modulus than C-PPG (≈10,000 Pa) (Figure 5C). Hydrogen bonds form between the HPAAm network and the primary network groups in the H-PPG, creating a cooperative, synergistic reinforcement that enhances shear strength and toughness. Polyacrylamide is known for its stretchability; as a result, over time and under thermal stress, this secondary network extends or unfolds, allowing the H-PPG to endure significant deformation, expand, and absorb water without tearing. The elastic modulus shows this trend, decreasing from about 45,000 Pa to roughly 10,000 Pa after 20 days of aging under thermal stress. Loss module ($G^{\prime \prime }$) decreases as aging time increases for C-PPG and H-PPG; this occurs because physical aging causes structural relaxation, where the material moves closer to a lower-energy, more stable, and denser equilibrium state, making the PPGs behave less like a viscous liquid and more like an elastic solid. Conversely, the loss modulus of P-PPG increases, making the material stiffer and more resistant to deformation and breaking.

**Figure 6:**
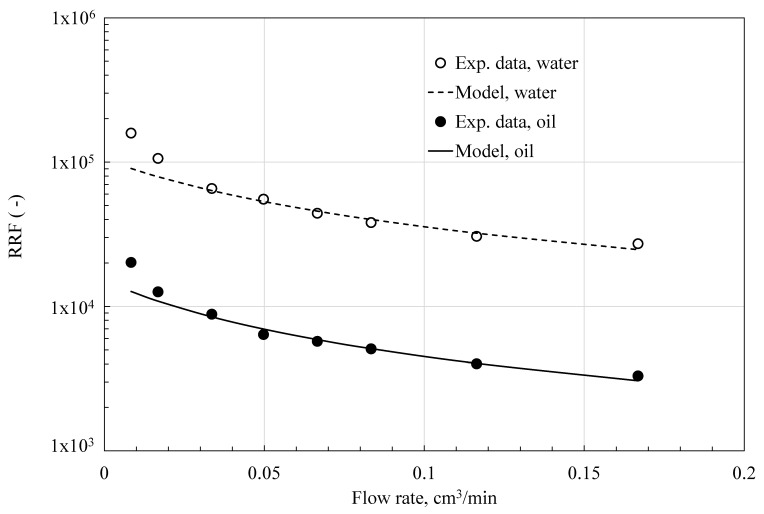
Data from experiments and Matías-Pérez’s model [12] of the residual resistance factor (RRF) as a function of flow rate, collected during C-PPG evaluation.

**Figure 7:**
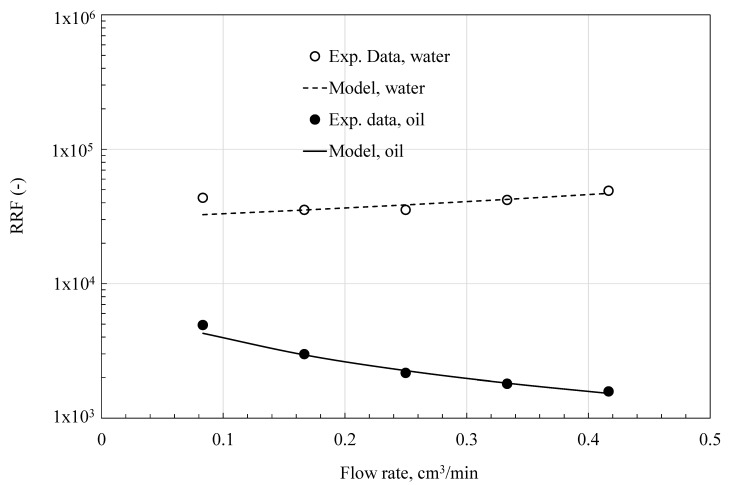
Data from experiments and Matías-Pérez’s model [12] of the residual resistance factor (RRF) as a function of flow rate, collected during P-PPG evaluation.

**Figure 8:**
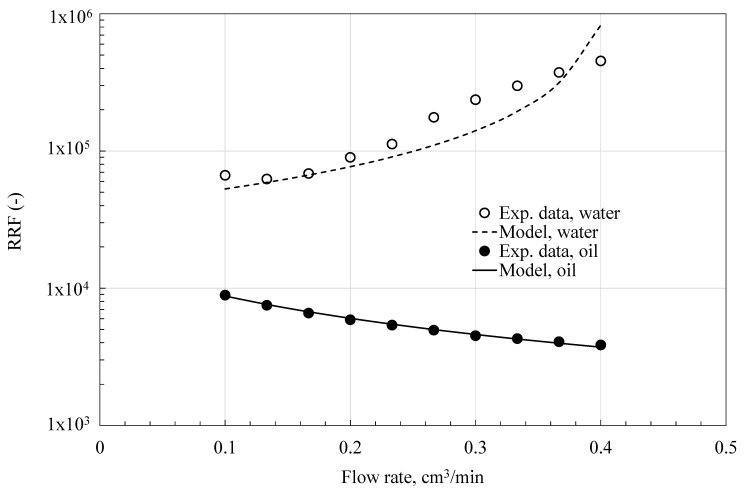
Data from experiments and Matías-Pérez’s model [12] of the residual resistance factor (RRF) as a function of flow rate, collected during H-PPG evaluation.

Figure 6, Figure 7 and Figure 8 show how residual resistance factors change with flow rate for water and oil phases in C-PPG, P-PPG, and H-PPG. Across the entire flow rate range, all PPGs have a higher RRF for water than for oil. In every case, oil RRF decreases with increasing flow rate, whereas water RRF varies across PPG types. In C-PPG, the water RRF shows typical behavior, decreasing with higher flow rates. Since this hydrogel washes out at a rate of 0.17 cm^3^/h, the assessment was performed in the low-shear-rate region. The water RRF in P-PPG remains roughly constant across the entire tested flow-rate range, whereas in H-PPG it increases with flow rate. P-PPG and H-PPG do not show degradation or washout over the evaluated flow-rate range. The observed RRF-flow rate relationship follows a power law model:

(6) $${RRF}_{f}=\frac{{\left[{∆P}_{f}\right]}_{a}}{{\left[{∆P}_{f}\right]}_{b}}=\frac{C_{a}\cdot q_{f}^{n}}{C_{b}{\cdot q}_{f}}=C\cdot q_{f}^{n-1}$$

where:

n = Particle behavior index, also known as the flow behavior index, which describes the PPG behavior: shear thinning (n < 1), hard sphere (n = 1), and shear thickening (n > 1).

C = Consistency index, which is related to gel strength.

Table 6 presents the particle behavior index at different flow rates. Hydrogel particles exhibit shear-thinning behavior during oil transport, thereby favoring oil flow (n < 1). Shear thinning during water flow is typical in PPG applications. As the water shear rate rises, the soft, water-swollen C-PPG deforms, elongates, and aligns with the flow direction (n = 0.417), exhibiting the usual behavior of conventional PPG [3,6,8,9,10,11,12]. Shear-thickening occurs in H-PPG (n = 2.5585) and P-PPG (n = 1.3561) as particles swell or form temporary hydroclusters, significantly increasing the flow resistance. Shear-thickening, often observed in systems with slower crosslinking kinetics, is explained by network reorganization under shear, in which ‘inactive’ intrachain crosslinks convert into ‘active’ interchain crosslinks, as it is manifested during the coil-to-extended transition of the HPAAm polymer inside the H-PPG structure [30]. Such intrachain-polymer crosslinks form through ionic interactions between aqueous cations and carboxylate moieties, as well as hydrogen bonding among its carboxylic acid moieties. Meanwhile, interchain crosslinks result from intermolecular interactions between the carboxylate group of the polymer chain and the surrounding system, including ions, clay, and the PPG main structure.

According to Matias-Perez’s model [12], fluid flow influences the evaluated deformable PPGs in various ways (Table 7). In C-PPG, the packed medium exhibits channeling behavior under water and oil flow. In both P-PPG and H-PPG, the medium exhibits compaction under water flow and channeling under oil flow. These last two PPGs display non-conventional behavior in water blockage, maintaining performance at high flow rates.

The initial permeability, $k_{0}$, is consistently higher during oil flow than during water flow. Due to the hydrophilic nature of PPGs, which underlies their use, they swell and block the initial free passage of water, thereby creating a barrier. C-PPG performs the worst because it requires relatively low pressure ($p_{1}$ = 3.69 psi) for water to begin channeling through the gel. P-PPG and H-PPG provide optimal performance as they compact effectively when water flows over them. However, for creating a barrier that permanently blocks water, H-PPG outperforms the others, because it requires a lower pressure ($p_{1}$ = 11.6 psi) than P-PPG ($p_{1}$ = 24.39 psi).

**Figure 9:**
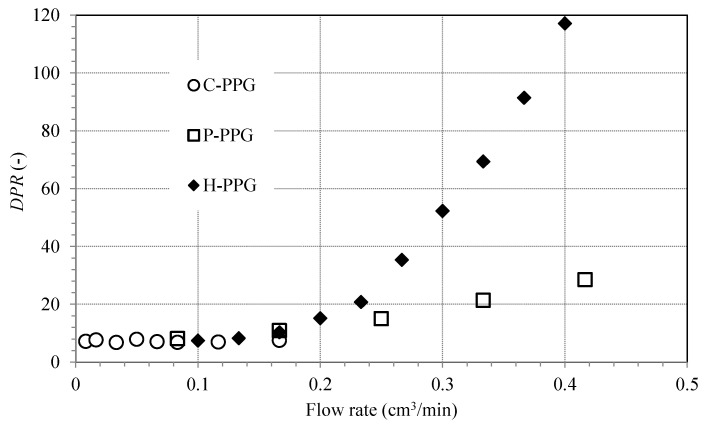
The behavior of the disproportionate permeability reduction across flow rates for C-PPG, P-PPG, and H-PPG.

The effectiveness of disproportionate permeability reduction (Figure 9) shows that all evaluated PPGs have significantly reduced the medium’s water permeability (high ${RRF}_{w}$) while allowing oil to pass through relatively unimpeded (${RRF}_{o}$ is lower). At low shear rates, corresponding to conditions deep within the reservoir where fluids flow slowly, all PPG types exhibit similar effectiveness up to 0.17 mL/min (DPR≈8). As flow rates rise, suggesting conditions nearer to the producer wells or within high-conductivity channels, C-PPG degrades, reducing its effectiveness and ability to block water flow. In contrast, P-PPG maintains its effectiveness as flow rate increases (DPR≈ 28 at 0.41 mL/min), whereas H-PPG performs better (DPR≈ 117 at 0.4 mL/min).

### 3.3. Computational Analysis

The following results quantitatively develop the outlined computational framework and are discussed in direct relation to the experimentally observed swelling, mechanical stability, and flow-response behavior of the different PPG systems.

#### 3.3.1. Reaction Energies for Obtaining Polymers That Compose the Hydrogels

**Figure 10:**
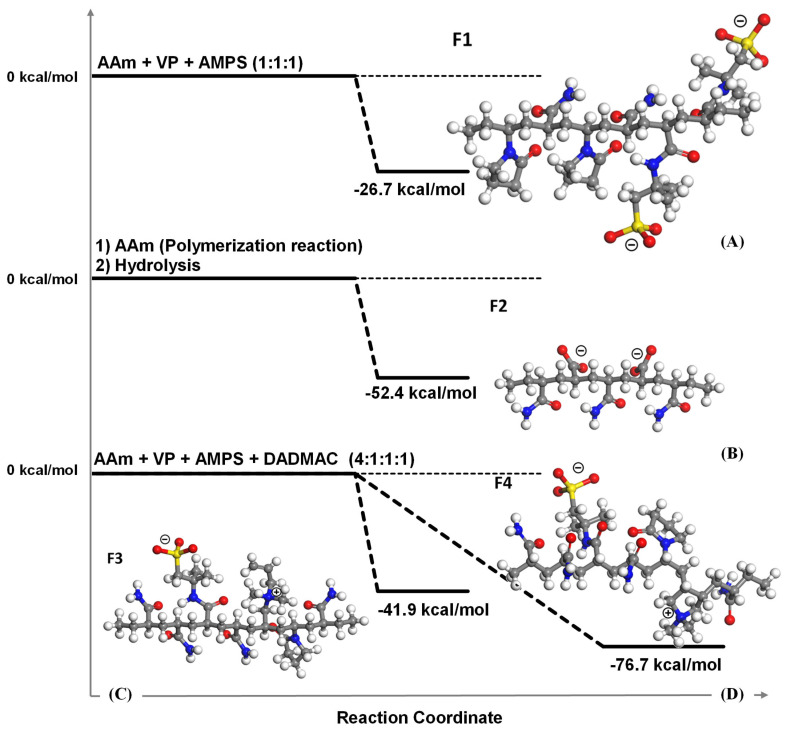
Schematic reaction paths for obtaining the model oligomers C-PPG (**A**) and HPAAm (**B**) composing the H-PPG hydrogel, and the ones P_A_-PPG (**C**) and P_B_-PPG (**D**) composing the P-PPG hydrogel.

The geometric optimization indicates that when AAm, VP, and AMPS react to form the oligomer C-PPG, a release of −26.7 kcal/mol occurs (Figure 10A). Similarly, the hydrolysis of AAm releases −52.4 kcal/mol to produce the oligomer HPAAm (Figure 10B). Additionally, the reaction energies for forming P_A_-PPG (Figure 10C) and P_B_-PPG (Figure 10D) from AAm, VP, AMPS, and DADMAC are −41.9 and −76.7 kcal/mol, respectively, indicating that the cyclic form of DADMAC results in a formation energy twice as high as that of the allylic form.

For clarity (Figure 10), the oligomeric fragments corresponding to C-PPG, HPAAm, P_A_-PPG, and P_B_-PPG are hereafter labeled as F1, F2, F3, and F4, respectively, and this notation is used consistently throughout this section.

#### 3.3.2. Electrostatic Complementarity and Electron-Density Topology in PPG Supramolecular Assemblies

**Figure 11:**
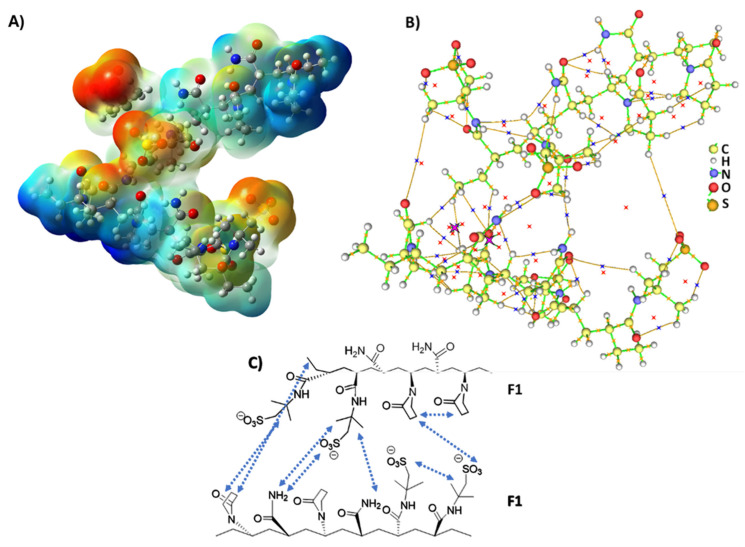
Distribution of the charge density and QTAIM analysis of the F1–F1 supramolecular complex. (**A**) Molecular electrostatic potential (ESP) mapped onto a constant electron-density isosurface. Regions of negative ESP are depicted in red and orange, whereas regions of less negative or positive ESP are shown in green and blue. (**B**) Electron-density critical points classified according to their topological indices: (3, −1) covalent bond critical points (orange stars), non-covalent bond critical points (blue stars), (3, +1) ring critical points (black-bordered pink stars), and (3, +3) cage critical points (red stars). Brown curved lines represent bond paths, i.e., trajectories of maximum electron density connecting neighboring nuclei and defining the interaction network. (**C**) Schematic representation of the most significant noncovalent intermolecular interactions within the supramolecular complex.

The ESP map of the complex formed by the F1–F1 dimer, which models the C-PPG hydrogel, displays a notably anisotropic charge distribution across the supramolecular surface (Figure 11A). Figures for the other studied species are available in the Supporting Information. The most negative regions are mainly around the sulfonate and amidic carbonyl oxygen atoms, indicating strong electron-rich domains. These areas correspond to intense negative electrostatic potential and therefore constitute preferential sites for electrophilic interactions and hydrogen-bond acceptor sites. In contrast, less negative or relatively positive regions are mainly distributed around hydrogen atoms bonded to electronegative centers and near positively polarized moieties, reflecting electron-deficient domains that can act as hydrogen-bond donors. The spatial separation between electron-rich and electron-poor regions across the oligomeric fragments indicates clear electrostatic complementarity within the complex. The magnitude and position of the potential extrema indicate that intermolecular association is directional and governed by well-defined regions of strong electrostatic contrast.

The bond critical points (3, −1) identified by the QTAIM analysis (Figure 11B) confirm the presence of conventional covalent interactions within each oligomer. Importantly, additional (3, −1) critical points are observed along intermolecular bond paths connecting atoms from different oligomeric fragments, demonstrating that the fragments are linked by genuine electron-density channels rather than by mere spatial proximity. This provides direct topological evidence of intermolecular interaction within the supramolecular assembly.

Additional topological features provide insights into the three-dimensional structure of the supramolecular complex. This includes ring critical points (3, +1), which arise from cyclic arrangements of bond paths, and isolated cage critical points (3, +3), associated with regions enclosed by multiple interacting atoms where the electron density reaches a local minimum. Together, these features indicate that the supramolecular complex is characterized by an interconnected network of intra- and intermolecular interactions, which are thoroughly described within the QTAIM framework.

Although QTAIM does not explicitly decompose interaction energies into electrostatic or dispersive components, it provides indirect evidence of dispersion-dominated contacts by identifying low-density (3, −1) bond critical points between weakly polarized atoms. In the F1–F1 supramolecular complex, several intermolecular bond paths linking nonpolar or weakly polar regions are associated with bond critical points of low electron density, characteristic of closed-shell interactions. These features are consistent with dispersion-driven contributions, where subtle electron-density accumulation along the intermolecular axis reflects the presence of London dispersion forces.

Specifically, the sulfonate groups act as primary electron-rich sites and provide the main electrostatic contributions, attracting relatively electron-deficient regions of the neighboring fragment, including polarized hydrogen atoms and positively biased functional groups. Additional stabilization arises from carbonyl oxygen atoms, which serve as secondary electron-rich sites capable of participating in hydrogen-bond-like interactions, as supported by electrostatic complementarity in the ESP map and the presence of intermolecular (3, −1) bond critical points.

Whereas the ESP map reflects the electrostatic landscape of the complex and thus primarily highlights charge complementarity, the QTAIM topology also reveals several low-density intermolecular bond paths between weakly polarized aliphatic regions of the pendant groups. Interactions involving the methyl groups of AMPS with the hydrocarbon backbone of the polymer, the aliphatic segment of VP, and even the partially exposed NH_2_ group of AAm are associated with weak bond critical points characteristic of closed-shell contacts. These features are consistent with dispersion-assisted stabilization between nonpolar or weakly polarized moieties. In parallel, more directional hydrogen-bonding interactions are observed between AMPS and both VP and AAm units, reinforcing the cooperative nature of the intermolecular network. Altogether, the supramolecular assembly arises from a synergistic interplay of strong electrostatic attractions, directional hydrogen bonding, and weaker but cumulative dispersive contacts distributed across the intermolecular interface.

ESP mapping and QTAIM critical points for the remaining supramolecular complexes are provided in the Supporting Information. In the F2–F2 dimer, representing the unswelled HPAAm polymeric matrix (Figure S1), consistent amide–amide supramolecular complementarity is observed, along with notable non-classical hydrogen bonding between carbonyl groups and the hydrocarbon backbone of adjacent oligomer chains.

In the F1–F2 dimer of the H-PPG hydrogel (Figure S2), stabilizing non-covalent interactions include amide–amide complementarity between the terpolymer C-PPG and PHPAM tectons, along with additional dispersive contacts involving the amide backbone. Moreover, partial electronic delocalization within the amide groups promotes favorable parallel alignment of some amide moieties, thereby enhancing dipolar and cooperative interactions across the interface. This higher density of non-covalent contacts in the mixed system, compared to the F1–F1 dimer of C-PPG, aligns with the enhanced supramolecular cohesion of the hydrogel network and may contribute to the observed syneresis behavior.

The F4–F4 dimer (Figure S3) appears to be a dominant interaction motif within the P-PPG polymer matrix. In this structure, the carbonyl and sulfonate groups create electron-rich regions, whereas the amidic protons form electron-deficient sites, establishing clear electrostatic polarization within the assembly. Analysis of the ESP distribution and QTAIM topology indicates that, although electrostatic and hydrogen-bonding interactions are present, dispersive contributions play a particularly significant role in contacts involving the aliphatic parts of the system. Specifically, weak intermolecular interactions are observed between the N, N-dimethylpyrrolidinium moieties and the methyl groups of AMPS, as well as with the polymer’s hydrocarbon backbone. In addition, hydrogen bonding between sulfonate or carbonyl oxygen atoms and amidic protons stabilizes the structure, and weaker C–H···O contacts involving methyl substituents further reinforce the supramolecular organization.

In the F3–F4 supramolecular complex (Figure S4), the sulfonate and carbonyl groups define electron-rich regions, whereas the allyl substituents and amidic protons form relatively electron-deficient domains, establishing a marked electrostatic polarization across the interface. Beyond electrostatic complementarity, the QTAIM analysis uncovers several weak intermolecular contacts between aliphatic regions of the system, including interactions between VP segments and the N, N-dimethylpyrrolidinium moieties of F4, as well as between the aliphatic portions of AMPS and the hydrocarbon backbone of F3. These low-density bond critical points are consistent with dispersion-assisted stabilization arising from the close packing of weakly polarized surfaces. Furthermore, weak C–H···O contacts are identified between sulfonate oxygen atoms and methyl or allylic hydrogen atoms, indicative of non-classical hydrogen bonding. An additional intermolecular S···S contact between spatially adjacent sulfonate groups of F3 and F4 is also detected through a low-density bond critical point. The small electron density and positive Laplacian at this point are characteristic of a weak closed-shell interaction, likely arising from spatial proximity and packing effects rather than from a directional chalcogen-bonding mechanism. Together, these interactions contribute to the cooperative stabilization of the F3–F4 supramolecular assembly.

#### 3.3.3. Flory-Huggins Interaction Parameter (χ_FH_) at Room Temperature and 92 °C for Each PPG

**Figure 12:**
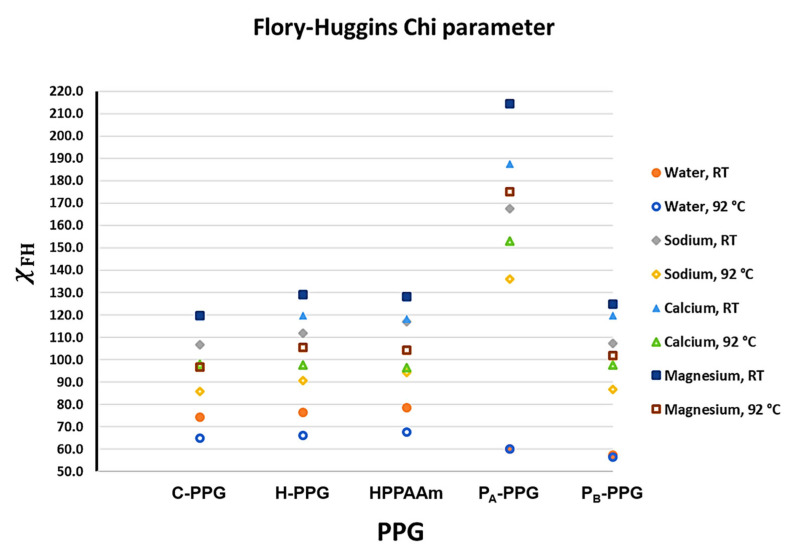
Temperature dependence of the Flory–Huggins interaction parameter ($\chi_{FH}$) for PPG matrices interacting with water and formation-water cations (Na^+^, Ca^2+^, Mg^2+^) at room temperature and 92 °C.

The calculated $\chi_{FH}$ values show a clear dependence of polymer–ion affinity on both cation valence and polymer architecture (Figure 12). Across all systems, interactions with water produce the lowest $\chi_{FH}$ values, indicating favorable thermodynamic compatibility and supporting the viability of swelling under formation-water conditions. In contrast, $\chi_{FH}$ values are significantly higher for divalent cations, particularly Mg^2+^, indicating reduced compatibility and a greater tendency for ionic perturbation of the polymer network. These trends align with the electrostatic landscape revealed by the ESP maps, in which highly negative sulfonate domains serve as preferential coordination sites for multivalent cations.

Among the matrices examined, P-PPG shows the lowest $\chi_{FH}$ values for water, indicating the highest intrinsic thermodynamic compatibility with the solvent. Notably, this behavior is largely independent of temperature across the studied range. The ESP analysis reveals well-defined electrostatic complementarity between polar functional groups and water, while the QTAIM topology identifies a dense network of intermolecular contacts, particularly in regions containing pyrrolidinium. Although segments bearing allyl pendant groups exhibit fewer directional interactions, likely due to steric constraints that increase interchain separation, the overall supramolecular organization remains cohesive. Despite its high-water affinity, P-PPG exhibits a relatively low experimental ESR value, suggesting slow water-uptake kinetics. This apparent discrepancy can be rationalized by the internally cooperative non-covalent framework revealed by QTAIM, which imposes structural constraints that limit rapid solvent diffusion. The combination of strong polymer–water compatibility and a cohesive supramolecular architecture is consistent with the reduced syneresis observed experimentally.

For H-PPG, the $\chi_{FH}$ values toward water fall between those of C-PPG and HPAAm, reflecting the mixed interaction landscape of the polymer blend. At room temperature, both PPG systems exhibit comparable water affinity, as indicated by comparable ESR values. However, marked differences emerge in their interactions with cationic species. The C-PPG matrix exhibits a stronger thermodynamic preference for cation coordination, as evidenced by lower $\chi_{FH}$ values with Na^+^ and, especially, with divalent cations. This behavior aligns with the ESP identification of highly exposed anionic sulfonate regions and with the QTAIM analysis, which shows a lower density of cooperative intermolecular interactions relative to H-PPG. Consequently, although C-PPG and H-PPG display similar water compatibility, the enhanced cation attraction in C-PPG promotes charge screening, network contraction, and more pronounced syneresis. This effect is directly reflected in the higher experimental SF values.

Overall, the Flory–Huggins analysis aligns with the microscopic organization revealed by ESP and QTAIM. Polymer matrices with cooperative electrostatic complementarity and dense supramolecular connectivity exhibit greater resilience to ionic perturbations, whereas systems with more exposed anionic sites or weaker intermolecular cohesion show heightened sensitivity to multivalent cations. Thus, the hydrogels’ macroscopic swelling (ESR) and syneresis (SF) behavior can be understood as an emergent consequence of their underlying electron-density topology and thermodynamic compatibility with water and dissolved ions.

The combined experimental and computational results demonstrate that the macroscopic flow-control performance of the modified PPGs emerges directly from their engineered supramolecular architectures. QTAIM and ESP analyses reveal that introducing cationic groups (P-PPG) and secondary sliding networks (H-PPG) increases the density, diversity, and cooperativity of non-covalent interactions, leading to mechanically resilient, shear-responsive networks. These microscopic features rationalize the experimentally observed trends in elastic modulus, syneresis resistance, and the residual resistance factor. Notably, the shear-thickening response and high DPR values measured for H-PPG at elevated flow rates are consistent with a supramolecular framework that can adaptively compact and dynamically reorganize under stress. Thus, theoretical analysis provides a molecular-level explanation for the non-conventional flow behavior observed experimentally, confirming that selective water blockage at high shear is an emergent consequence of suprastructural design rather than just macroscopic formulation.

## 4. Conclusions

PPG’s application has been greatly broadened to address more severe stress conditions, primarily due to the enhanced density of supramolecular interactions. These advanced hydrogels employ non-covalent, multi-component networks to achieve structural stability and mechanical strength.

Two key strategies were implemented to enhance the suprastructural network of a composite PPG (C-PPG): doping with cationic moieties and incorporating a second anionic sliding macromolecule, thereby creating both a polyampholyte (P-PPG) and a hybrid PPG (H-PPG). These modifications not only ensure disproportionate permeability reduction (DPR) under high-flow-rate conditions but also help the PPGs to withstand harsh environments with extreme temperatures, salinity, and water hardness.

The C-PPG exhibits typical adverse behavior as the water flow rate increases: the water residual resistance factor (RRF) decreases in a power-law manner (n < 1). At the same time, its ability to promote DPR is limited by mechanical performance. Conversely, the proposed P-PPG and H-PPG exhibit improved water RRF and DPR trends in the high-flow-rate region, where the medium exhibits shear-thickening behavior (n > 1), thereby enhancing water blocking. These are consequences of swollen PPGs’ response to stress: C-PPG geometry compresses, allowing water channeling, whereas P-PPG and H-PPG maintain and expand their geometry, respectively, resulting in media compaction that favors water retention. In all cases, during oil flow, the packed media behaves as a shear-thinning medium (n < 1), promoting oil flow as the flow rate increases.

Interactions with cationic moieties enhance the mechanical strength of P-PPG, making it the strongest gel evaluated. This leads to a lower swelling ratio and a higher, more stable elastic modulus. The consistent syneresis factor over time indicates that this structure remains stable across thermal, salinity, and hardness conditions. Experimental results show that P-PPG preferentially reduces water flow, as evidenced by the water DPR in high-flow regions increasing from 8 to 28, with 8 serving as the C-PPG baseline.

The dispersed anionic polymer, the basis of the second network in H-PPG, increases the density of interactions arising from its charge. The observed improvements result from the structural expansion of H-PPG, driven by the behavior of dispersed polymers. Relaxation of polymer chains, caused by increased thermal stress and the expansion of their coil conformation under high shear conditions, results in greater solvation and larger H-PPG particles. When swollen particles are exposed to prolonged thermal and salinity stress, they expand while maintaining their elastic modulus. Tests reveal a strong ability of H-PPG to preferentially reduce water flow over oil, with DPR increasing from 8 to 117 in the high-flow-rate zone.

Proposed improvements allow PPGs to endure high flow rates while effectively blocking water over oil, which is essential for applications near producer wells and in high-conductivity channels within oil reservoirs.

Beyond demonstrating improved performance, this work establishes a multiscale design strategy in which supramolecular engineering is guided and validated by quantum-chemical and thermodynamic analyses. By explicitly linking electron-density topology, polymer–ion compatibility, and macroscopic flow behavior, the study provides a predictive framework for developing next-generation PPG systems capable of selective water blockage under extreme reservoir conditions. From a molecular perspective, the superior performance of P-PPG and H-PPG is attributed to the formation of a highly cooperative, topologically connected supramolecular network, as revealed by QTAIM and polymer–ion thermodynamic analysis, which enables efficient stress redistribution, adaptive compaction, and resistance to washout under high-flow conditions.

## Figures and Tables

**Table 1:** Chemical composition of production brine.

Ion	Production Brine (mg/L)
Na^+^	27,150.78
Ca^+2^	4616.00
Mg^+2^	495.92
Fe^+2^	0.75
Cl^−^	51,200.00
SO_4_^−2^	40.00
HCO_3_^−^	439.20
TDS	83,942.65

**Table 2:** Summary of the PPGs components addressed in this work.

Component	Preformed Particle Gel [g]		
	C-PPG	P-PPG	H-PPG
Acrylamide (AAm)	7.773	16.280	7.773
Vinylpyrrolidone (VP)	12.153	6.363	12.153
Sodium 2-acrylamido-2-methylpropane sulfonic (AMPSNa)	25.073	13.127	25.073
Diallyldimethylammonium Chloride (DADMAC)		9.229	
N,N′-Methylenebis(acrylamide) (MBA)	0.750	0.375	0.750
40% Hydrolyzed Polyacrylamide (HPAAm)			3.0
Modified Bentonite (MB)	60.0	60.0	15.0
Ammonium persulfate (APS)	0.150	0.075	0.225
N,N,N′,N′-Tetramethyl ethylenediamine (TEMED)	0.075	0.045	

**Table 3:** Assignment of carbon chemical shifts in H-PPG and P-PPG.

Signal Type	Chemical Shift (ppm)		Signal Assignment
	H-PPG	P-PPG	
-C=O	181.9	182.9	AAm
-C=O	181.4	182.6	AMPSNa
-C=O	180.7	182.1	VP
	64.5	64.8	AMPSNa
-CH_2_-	61.1	60.6	AMPSNa
-C-	56.4	56.3	AMPSNa
-CH_2_-	45.9	45.4	AMPSNa, VP, AAm
-CH_2_-	35.6	35.9	VP, AAm
-CH_3_-	31.9	32.2	AMPSNa
-CH_2_-	22.2	22.0	VP
-CH_3_-	-	53.9	DADMAC
-CH_2_-	-	70.1	
-CH_2_-	-	74.0	
=CH_2_	-	129.2	
-CH-	-	133.2	

**Table 4:** Identification of the bands associated with the functional groups in the terpolymers H-PPG and P-PPG.

Functional Groups	Wave Number υ (cm^−1^)		Observations
	P-PPG	H-PPG	
-NH/-NH_2_	3336	3405, 3325	Amide
-CH_2_, -CH_3_	3196, 2929	2976, 2936	Methylene and methyl
-CO-N-(R)_2_	1652	1652	Carbonyl of amide
-C(CH_3_)_2_-	1184	1184	Geminal methyl
-S=O	1038	1039	Sulfonate

**Table 5:** The equilibrium swelling ratio (ESR) and syneresis factor (SF) for the behavior of preformed particle gel (PPG) in production water.

PPG Type	ESR (−)	SF (%)
Composite (C-PPG)	11.20	0.2
Polyampholite (P-PPG)	4.39	0.36
Hybrid (H-PPG)	11.22	−53.44

**Table 6:** Flow behavior (***n***) and consistency (C) indexes, from the fitted power-law model, describing the dependence of the residual resistance factor (RRF) on flow rate for the synthesized PPGs.

PPG	Water		Oil	
	*n*	C	*n*	C
C-PPG	0.417	9159.4	0.398	1110.2
P-PPG	1.3561	63,279.0	0.285	824.7
H-PPG	2.5585	1,492,020.6	0.392	2202.0

**Table 7:** Adjusted parameters of Matías-Pérez’s model [12] that govern PPG flow behavior.

	Water			Oil		
Type of PPG	$k_{0}$ [D]	$p_{1}$ [psi]	Flow Behavior	$k_{0}$ [D]	$p_{1}$ [psi]	Flow Behavior
C-PPG	2.80	3.69	Channeling	19.32	0.44	Channeling
P-PPG	9.70	24.39	Compaction	38.15	0.54	Channeling
H-PPG	7.30	11.6	Compaction	18.38	1.22	Channeling

## Data Availability

The raw data supporting the conclusions of this article will be made available upon request by the authors.

## Supplementary Materials

The following supporting information can be downloaded at: https://www.mdpi.com/article/10.3390/polym18070850/s1, Figure S1: Electronic and QT-AIM analysis for the selected example of dimer F2-F2, representing the E-PPG showing (A) the ESP map depicting the range in the distribution of charge density (in e-/Å), (B) Non-covalent interactions calculated in the QT-AIM analysis of the dimer and (C) Schematic representation of the most relevant non-covalent interactions giving rise to the suprastructure of F2-F2 dimer; Figure S2: Electronic and QT-AIM analysis for the selected example of dimer F2-F2, representing the E-PPG showing (A) the ESP map depicting the range in the distribution of charge density (in e-/Å), (B) Non-covalent interactions calculated in the QT-AIM analysis of the dimer and (C) Schematic representation of the most relevant non-covalent interactions giving rise to the suprastructure of F2-F2 dimer; Figure S3: Electronic and QT-AIM analysis for the selected example of dimer F4-F4, representing the E-PPG showing (A) the ESP map depicting the range in the distribution of charge density (in e-/Å), (B) Non-covalent interactions calculated in the QT-AIM analysis of the dimer and (C) Schematic representation of the most relevant non-covalent interactions giving rise to the suprastructure of F4-F4 dimer; Figure S4: Electronic and QT-AIM analysis for the selected example of dimer F3-F4, representing the E-PPG showing (A) the ESP map depicting the range in the distribution of charge density (in e-/Å), (B) Non-covalent interactions calculated in the QT-AIM analysis of the dimer and (C) Schematic representation of the most relevant non-covalent interactions giving rise to the suprastructure of F3-F4 dimer; Figure S5: Overlapping spectra of ^13^C NMR CP/MAS: terpolymer H-PPG (line purple) and homopolymer polyacrylamide (line green), acquired to 150 MHz; Figure S6: Overlapping spectra of ^13^C NMR CP/MAS: terpolymer H-PPG (line purple) and homopolymer polyvinylpyrrolidone (line green), acquired to 150 MHz; Figure S7: Overlapping spectra of ^13^C NMR CP/MAS: terpolymer H-PPG (line purple) and homopolymer polyNaAMPS (line green), acquired at 150 MHz; Figure S8: Overlapping spectra of ^13^C NMR CP/MAS: terpolymer P-PPG (line purple) and homopolymer polyacrylamide (line green), acquired at 150 MHz; Figure S9: Overlapping spectra of 13C NMR CP/MAS: terpolymer P-PPG (line purple) and homopolymer polyvinylpyrrolidone (line green), acquired at 150 MHz; Figure S10: Overlapping spectra of ^13^C NMR CP/MAS: terpolymer P-PPG (line purple) and homopolymer polyNaAMPS (line green), acquired at 150 MHz; Figure S11: Overlapping spectra of FTIR-ATR: terpolymer H-PPG (line purple) and homopolymer polyacrylamide (line green); Figure S12: Overlapping spectra of FTIR-ATR: terpolymer H-PPG (line purple) and homopolymer polyvinylpyrrolidone (line green); Figure S13: Overlapping spectra of FTIR-ATR: terpolymer H-PPG (line purple) and homopolymer polyNaAMPS (line green); Figure S14: Overlapping spectra of FTIR-ATR: terpolymer P-PPG (line purple) and homopolymer polyacrylamide (line green); Figure S15: Overlapping spectra of FTIR-ATR: terpolymer P-PPG (line purple) and homopolymer polyvinylpyrrolidone (line green); Figure S16: Overlapping spectra of FTIR-ATR: terpolymer P-PPG (line purple) and homopolymer polyNaAMPS (line green); Figure S17. DSC curves of the terpolymer Poly-AAm, Poly-VP, Poly-PAMPS, and P-PPG; Figure S18. Comparison of TGA curves of the terpolymer Poly-AAm, Poly-VP, Poly-PAMPS, and P-PPG; Figure S19. DSC curves of the terpolymer Poly-AAm, Poly-VP, Poly-PAMPS, and H-PPG; Figure S20. TGA curves of the terpolymer Poly-AAm, Poly-VP, Poly-PAMPS, and H-PPG; Figure S21. ESEM micrographs of the P-PPG and H-PPG terpolymers reveal pore structures resulting from swelling in desionized water; Figure S22. ESEM micrographs of the P-PPG and H-PPG terpolymers reveal pore structures resulting from swelling in formation brine; Figure S23. Evolution of the linear viscoelastic region of C-PPG, during aging with formation brine at 130 °C; Figure S24. Evolution of the linear viscoelastic region of P-PPG, during aging with formation brine at 130 °C; Figure S25. Evolution of the linear viscoelastic region of H-PPG, during aging with formation brine at 130 °C; Table S1: Matrix for the calculated combinations forming E-PPG and HPAAm. Energies are given in Hartrees and relative to the reagents in their polymerization reaction. Lowest energy fragments are highlighted; Table S2: Matrix for the calculated combinations forming E-PPG and HPAAm. Energies are given in Hartrees and relative to the reagents in their polymerization reaction. Lowest energy fragments are highlighted; Table S3: Tabulated results for the Flory-Huggins Chi parameter calculated at room temperature (RT) and 92 °C for the studied PPG fragments; Table S4: Assignment of the signals of the spectra ^13^C NMR of the polymers: H-PPG, PAM, PVP, and PNaAMPS; Table S5: Assignation of the signals of the spectra ^13^C NMR of the polymers: P-PPG, PAM, PVP, and PNaAMPS; Table S6: Associated bands to the functional groups present in the polymers H-PPG, PAM, PVP, and PNaAMPS; Table S7: Associated bands to the functional groups present in the polymers P-PPG, PAM, PVP and PNaAMPS; Table S8: Results of elemental analysis of terpolymers H-PPG and P-PPG; Table S9: Elemental analysis percentages of normalized H-PPG and P-PPG Samples; Table S10: Empirical formulas of samples of terpolymers H-PPG and P-PPG; Table S11: Elemental compositions of the terpolymers H-PPG and P-PPG. Thermal JEOL; Table S12. Compositional range for P-PPG optimization; Table S13. Compositional range for H-PPG optimization. Refs. [44,45] cited in Supplementary Materials.
