# Supplementary material for: Shear-Responsive Supramolecular Preformed Particle Gel: Tailoring Network Architectures for Selective Water Blocking
Source: Polymers (Basel). 2026 Mar 31;18(7):850. doi: 10.3390/polym18070850 (PMC13074826; doi:10.3390/polym18070850)
Supplement: Supplementary file 1 [file polymers-18-00850-s001.zip › polymers-4202570-supplementary.pdf]

Supporting Information

Shear-Responsive Supramolecular Preformed Particle Gel: Tailoring Network Architectures for Selective Water Blocking.

Supporting Information Contents

	Page
Computational methodology	S3
Spectroscopic characterization of terpolymers by ^{13}C NMR	S10
Spectroscopic characterization of terpolymers by FTIR-ATR	S15
Characterization of terpolymers by Elemental Analysis	S20
Characterization of terpolymers by DSC and TGA	S22
Characterization of terpolymers by Environmental Scanning Electron Microscope (ESEM)	S25
Optimization of components of P-PPG and H-PPG.	S28.
Evolution of the linear viscoelastic region of PPGs, during aging with formation brine at 130 °C	S29

Computational methodology:

For all studied species, isotactic and atactic species were neglected. The former is due to stability and steric hindrance, and the latter is due to the unpredictability of formation and study. Therefore, the combinatorial grid was constructed solely from syndiotactic polymer segments.

E-PPG:

A polymer segment consisting of 6 monomers was considered, with the reaction stoichiometry, yielding 2 units of each species in the terpolymer. The combinatorial grid consisted of 92 possible segments. From these, stereochemistry was considered, and enantiomeric segments were neglected, as they would exhibit the same thermodynamic properties, leaving 48 fragments to analyze.

H-PPG:

In addition to the polymeric segment from the E-PPG, under the same criteria, HPAAm segments were calculated with 5 acrylamide units. To account for the 40% degree of hydrolysis, 3 amides and 2 carboxylates were designated as hanging groups for the 4 segments in the corresponding combinatorial grid.

P-PPG:

With a 4:1:1:1 stoichiometry for AAm, VP, AMPS, and DADMAC, a combinatorial grid with this proportion was determined, yielding 210 polymeric segments. To reduce the necessity of studying the whole combinatorial grid, the probabilistic and chemically intuitive criteria that the segments with four or three consecutive AAms were less probable to form were applied. For polymer segments with 4 consecutive AAm monomers, there is only 3-7% chance of finding them in the structure. For the segments with 3 AAm consecutive monomers, this chance increases considerably to 11-40%, but the fragments with 2 AAm consecutive monomers can be formed with a probability of 28-60%. Therefore, this criterion serves to study the most probable, most abundant structural motif in the hydrogel's polymeric matrix. After this, the stereochemistry criterion of eliminating the enantiomeric segments was also applied. This provided a final combinatorial grid of 39 polymer segments. But, as has been reported, the DADMAC fragment can be found in its more stable N,N-dimethylpyrrolidinium form, as well as in the less favored structure with a dangling allyl group. This yielded another 39 fragments to study.

Table S1. Matrix for the calculated combinations forming E-PPG and HPAAm. Energies are given in Hartrees and relative to the reagents in their polimerization reaction. Lowest energy fragments are highlighted.

Reagent	AAm	VP	AMPS	OH	NH3		
Energy PM6 (Ha) =	-0.020573	0.030187	-0.354847	-0.244867	0.000108		
			E-PPG explored fragment combinations				
							Energy PM6 (Ha)
1	AAm	AAm	VP	VP	AMPS	AMPS	-0.730797
2	AAm	AAm	VP	AMPS	VP	AMPS	-0.721844
3	AAm	AAm	VP	AMPS	AMPS	VP	-0.721528
4	AAm	AAm	AMPS	VP	VP	AMPS	-0.725552
5	AAm	AAm	AMPS	VP	AMPS	VP	-0.721422
6	AAm	AAm	AMPS	AMPS	VP	VP	-0.729913
7	AAm	VP	AAm	VP	AMPS	AMPS	-0.727553
8	AAm	VP	AAm	AMPS	VP	AMPS	-0.726313
9	AAm	VP	AAm	AMPS	AMPS	VP	-0.727107
10	AAm	VP	VP	AAm	AMPS	AMPS	-0.731872
11	AAm	VP	VP	AMPS	AAm	AMPS	-0.727200
12	AAm	VP	VP	AMPS	AMPS	AAm	-0.731759
13	AAm	VP	AMPS	AAm	VP	AMPS	-0.726982
14	AAm	VP	AMPS	AAm	AMPS	VP	-0.725692
15	AAm	VP	AMPS	VP	AAm	AMPS	-0.725283
16	AAm	VP	AMPS	VP	AMPS	AAm	-0.723689
17	AAm	VP	AMPS	AMPS	AAm	VP	-0.724774
18	AAm	VP	AMPS	AMPS	VP	AAm	-0.723775
19	AAm	AMPS	AAm	VP	VP	AMPS	-0.727187
20	AAm	AMPS	AAm	VP	AMPS	VP	-0.725708
21	AAm	AMPS	AAm	AMPS	VP	VP	-0.722526
22	AAm	AMPS	VP	AAm	VP	AMPS	-0.727349
23	AAm	AMPS	VP	AAm	AMPS	VP	-0.720383
24	AAm	AMPS	VP	VP	AAm	AMPS	-0.731950
25	AAm	AMPS	VP	VP	AMPS	AAm	-0.727231
26	AAm	AMPS	VP	AMPS	AAm	VP	-0.724486
27	AAm	AMPS	AMPS	AAm	VP	VP	-0.722101
28	AAm	AMPS	AMPS	VP	AAm	VP	-0.724126
29	VP	AAm	AAm	VP	AMPS	AMPS	-0.728967
30	VP	AAm	AAm	AMPS	VP	AMPS	-0.724566
31	VP	AAm	AAm	AMPS	AMPS	VP	-0.727470
32	VP	AAm	VP	AAm	AMPS	AMPS	-0.732956
33	VP	AAm	VP	AMPS	AAm	AMPS	-0.726197
34	VP	AAm	AMPS	AAm	VP	AMPS	-0.722911
35	VP	AAm	AMPS	AAm	AMPS	VP	-0.725332
36	VP	AAm	AMPS	VP	AAm	AMPS	-0.725744
37	VP	AAm	AMPS	AMPS	AAm	VP	-0.727798
38	VP	VP	AAm	AAm	AMPS	AMPS	-0.732594
39	VP	VP	AAm	AMPS	AAm	AMPS	-0.728453
40	VP	VP	AMPS	AAm	AAm	AMPS	-0.726716
41	VP	AMPS	AAm	AAm	VP	AMPS	-0.723688
42	VP	AMPS	AAm	AAm	AMPS	VP	-0.715030
43	VP	AMPS	AAm	VP	AAm	AMPS	-0.725626
44	VP	AMPS	VP	AAm	AAm	AMPS	-0.727181
45	AMPS	AAm	AAm	VP	VP	AMPS	-0.730869
46	AMPS	AAm	VP	AAm	VP	AMPS	-0.728240
47	AMPS	AAm	VP	VP	AAm	AMPS	-0.732563
48	AMPS	VP	AAm	AAm	VP	AMPS	-0.732667
			HPAAm explored fragment combinations				
49	Am	Am	COO	Am	COO		-0.673919
50	Am	Am	COO	COO	Am		-0.673927
51	Am	COO	Am	Am	COO		-0.672192
52	Am	COO	Am	COO	Am		-0.676364

Table S2. Matrix for the calculated combinations forming E-PPG and HPAAm. Energies are given in Hartrees and relative to the reagents in their polymerization reaction. Lowest energy fragments are highlighted.

Reagent	AAm	VP	AMPS	DADMAC					
Energy PM6 (Ha) =	-0.020573	0.030187	-0.354847	0.36091					
								DADMAC (hanging allyl)	DADMAC (pyrrolidinium)
								Energy PM6 (Ha)	Energy PM6 (Ha)
1	AAm	AAm	VP	AAm	DADMAC	AAm	AMPS	-0.111229	-0.155158
2	AAm	AAm	VP	AAm	DADMAC	AMPS	AAm	-0.109935	-0.155702
3	AAm	AAm	VP	AAm	AMPS	AAm	DADMAC	-0.112271	-0.150901
4	AAm	AAm	VP	AAm	AMPS	DADMAC	AAm	-0.110370	-0.155062
5	AAm	AAm	VP	DADMAC	AAm	AMPS	AAm	-0.104918	-0.152395
6	AAm	AAm	VP	AMPS	AAm	DADMAC	AAm	-0.112622	-0.149188
7	AAm	AAm	DADMAC	AAm	VP	AAm	AMPS	-0.104554	-0.154918
8	AAm	AAm	DADMAC	AAm	VP	AMPS	AAm	-0.106075	-0.148228
9	AAm	AAm	DADMAC	AAm	AMPS	AAm	VP	-0.110771	-0.14972
10	AAm	AAm	DADMAC	AAm	AMPS	VP	AAm	-0.110810	-0.148926
11	AAm	AAm	DADMAC	VP	AAm	AMPS	AAm	-0.103239	-0.152008
12	AAm	AAm	DADMAC	AMPS	AAm	VP	AAm	-0.106783	-0.156899
13	AAm	AAm	AMPS	AAm	VP	AAm	DADMAC	-0.108918	-0.152806
14	AAm	AAm	AMPS	AAm	VP	DADMAC	AAm	-0.110513	-0.168313
15	AAm	AAm	AMPS	AAm	DADMAC	AAm	VP	-0.107439	-0.155215
16	AAm	AAm	AMPS	AAm	DADMAC	VP	AAm	-0.112763	-0.148499
17	AAm	AAm	AMPS	VP	AAm	DADMAC	AAm	-0.11188	-0.148540
18	AAm	AAm	AMPS	DADMAC	AAm	VP	AAm	-0.107879	-0.153098
19	AAm	VP	AAm	AAm	DADMAC	AAm	AMPS	-0.109813	-0.154696
20	AAm	VP	AAm	AAm	DADMAC	AMPS	AAm	-0.112332	-0.154897
21	AAm	VP	AAm	AAm	AMPS	AAm	DADMAC	-0.111159	-0.148099
22	AAm	VP	AAm	AAm	AMPS	DADMAC	AAm	-0.103662	-0.151207
23	AAm	VP	AAm	DADMAC	AAm	AAm	AMPS	-0.112058	-0.155361
24	AAm	VP	AAm	DADMAC	AAm	AMPS	AAm	-0.110887	-0.151020
25	AAm	VP	AAm	AMPS	AAm	AAm	DADMAC	-0.106672	-0.154074
26	AAm	VP	AAm	AMPS	AAm	DADMAC	AAm	-0.112430	-0.155601
27	AAm	VP	DADMAC	AAm	AAm	AMPS	AAm	-0.112109	-0.151772
28	AAm	VP	AMPS	AAm	AAm	DADMAC	AAm	-0.106184	-0.149603
29	AAm	DADMAC	AAm	AAm	VP	AAm	AMPS	-0.112178	-0.155627
30	AAm	DADMAC	AAm	AAm	VP	AMPS	AAm	-0.107183	-0.148483
31	AAm	DADMAC	AAm	AAm	AMPS	AAm	VP	-0.110114	-0.149553
32	AAm	DADMAC	AAm	VP	AAm	AAm	AMPS	-0.109388	-0.151879
33	AAm	DADMAC	AAm	VP	AAm	AMPS	AAm	-0.111052	-0.149800
34	AAm	DADMAC	AAm	AMPS	AAm	AAm	VP	-0.107432	-0.152439
35	AAm	DADMAC	VP	AAm	AAm	AMPS	AAm	-0.108422	-0.151980
36	AAm	AMPS	AAm	AAm	VP	AAm	DADMAC	-0.110607	-0.151039
37	AAm	AMPS	AAm	AAm	DADMAC	AAm	VP	-0.105982	-0.148769
38	AAm	AMPS	AAm	VP	AAm	AAm	DADMAC	-0.106582	-0.150623
39	AAm	AMPS	AAm	DADMAC	AAm	AAm	VP	-0.108362	-0.147794

Table S3. Tabulated results for the Flory-Huggins Chi parameter calculated at room temperature (RT) and 92 °C for the studied PPG fragments.

		$\chi_{F\text{-}H}$							
		H_2O		Na^+		Ca^{2+}		Mg^{2+}	
**PPG**		**RT**	**92 °C**	**RT**	**92 °C**	**RT**	**92 °C**	**RT**	**92 °C**
**PPG-E**	**1**	74.3	65.0	106.7	85.8	120.3	97.8	119.6	96.7
**PPG-H**	**2**	76.3	66.2	111.9	90.7	119.6	97.5	129.2	105.5
**HPAAm**	**3**	78.5	67.5	116.9	94.4	118.3	96.3	128.2	104.3
**PPG-P$_A$**	**4**	60.0	60.0	167.7	136.0	187.5	152.9	214.5	175.1
**PPG-P$_B$**	**5**	57.2	56.3	107.3	86.7	119.8	97.6	125.0	102.0

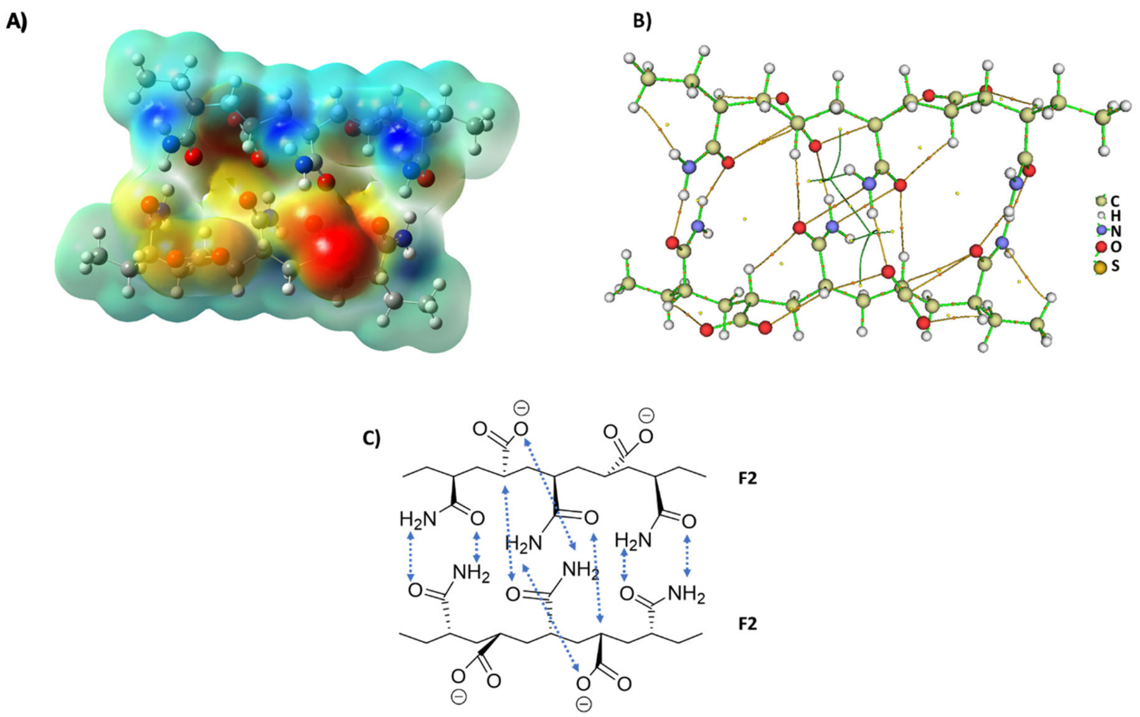

Figure S1. Electronic and QT-AIM analysis for the selected example of dimer **F2-F2**, representing the E-PPG showing A) the ESP map depicting the range in the distribution of charge density (in e^-/Å), B) Non-covalent interactions calculated in the QT-AIM analysis of the dimer and (C) Schematic representation of the most relevant non-covalent interactions giving rise to the suprastructure of **F2-F2** dimer.

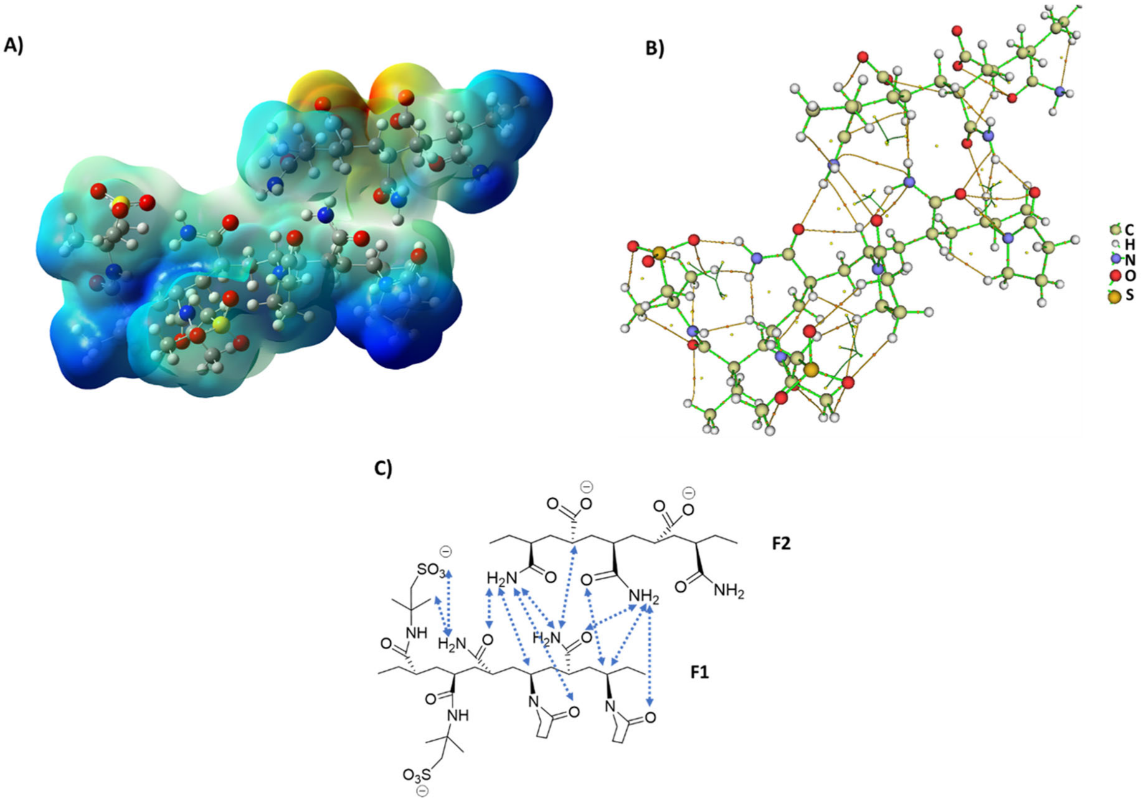

Figure S2. Electronic and QT-AIM analysis for the selected example of dimer **F1-F2**, representing the E-PPG showing A) the ESP map depicting the range in the distribution of charge density (in $e^-/Å$), B) Non-covalent interactions calculated in the QT-AIM analysis of the dimer and (C) Schematic representation of the most relevant non-covalent interactions giving rise to the suprastructure of **F1-F2** dimer.

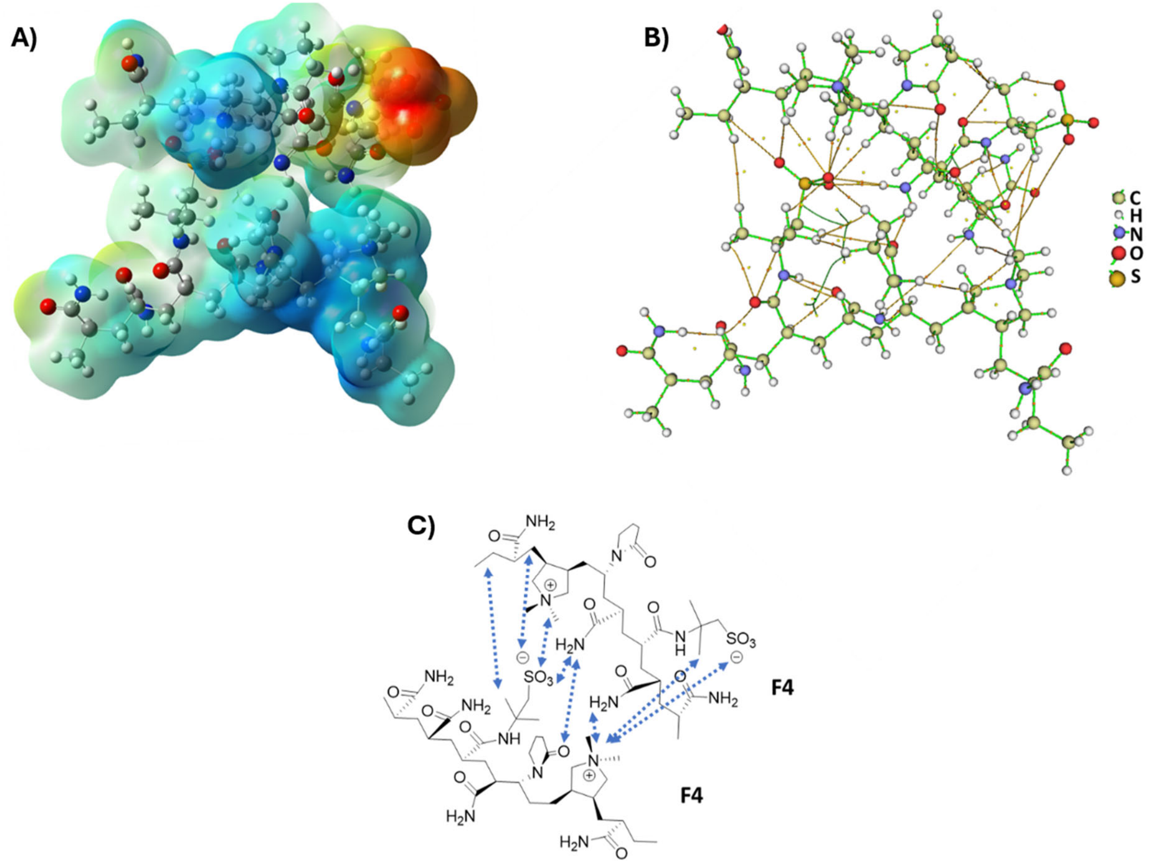

Figure S3. Electronic and QT-AIM analysis for the selected example of dimer **F4-F4**, representing the E-PPG showing A) the ESP map depicting the range in the distribution of charge density (in e^-/Å), B) Non-covalent interactions calculated in the QT-AIM analysis of the dimer and (C) Schematic representation of the most relevant non-covalent interactions giving rise to the suprastructure of **F4-F4** dimer.

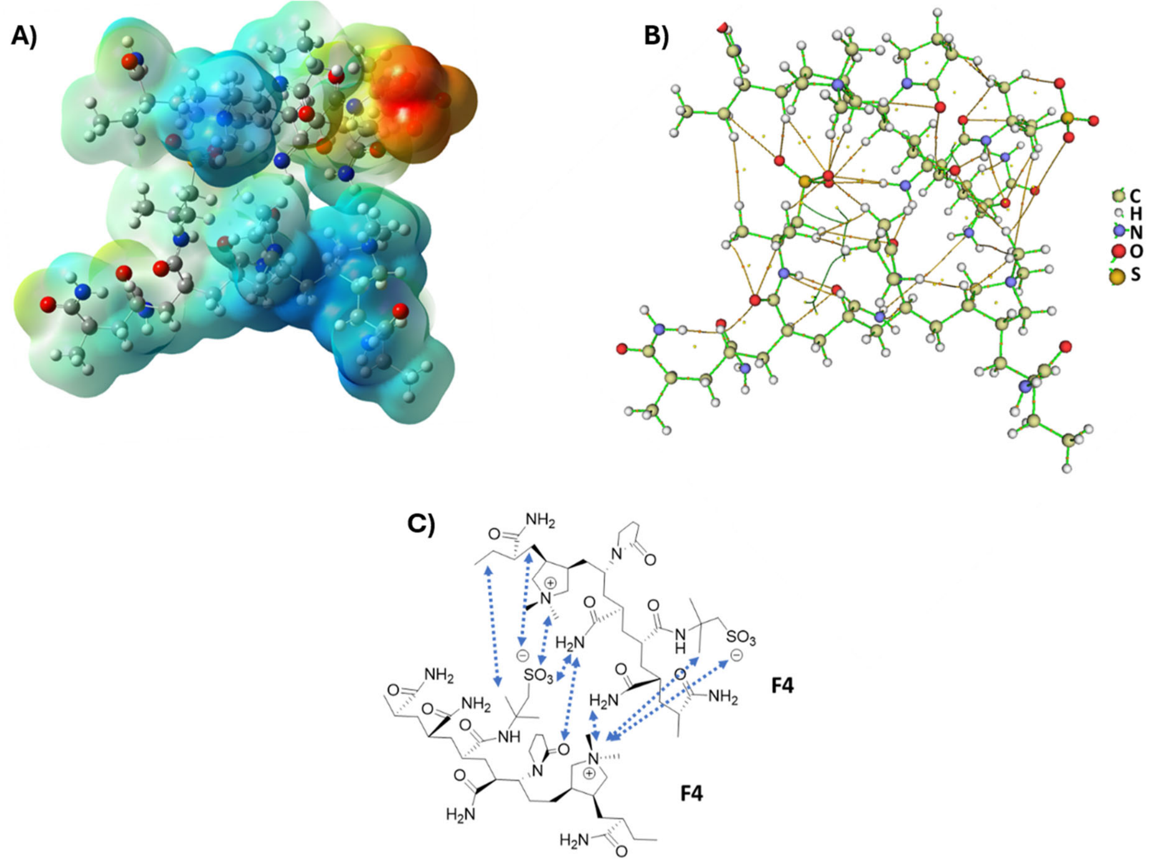

Figure S4. Electronic and QT-AIM analysis for the selected example of dimer **F3-F4**, representing the E-PPG showing A) the ESP map depicting the range in the distribution of charge density (in $e^{-}/Å$), B) Non-covalent interactions calculated in the QT-AIM analysis of the dimer and (C) Schematic representation of the most relevant non-covalent interactions giving rise to the suprastructure of **F3-F4** dimer.

Spectroscopic characterization of terpolymers by ¹³C NMR.

Compound: H-PPG

Figures S5-S7 show the overlapping ^{13}C NMR CP/MAS spectra of terpolymer H-PPG (line purple) with polyacrylamide, polyvinylpyrrolidone, and polyNaAMPS (line green), respectively. The signals generated by each homopolymer coincide with the chemical shifts of the signals present in the spectrum of terpolymer H-PPG. Table S4 describes the signals present in the overlapping spectra.

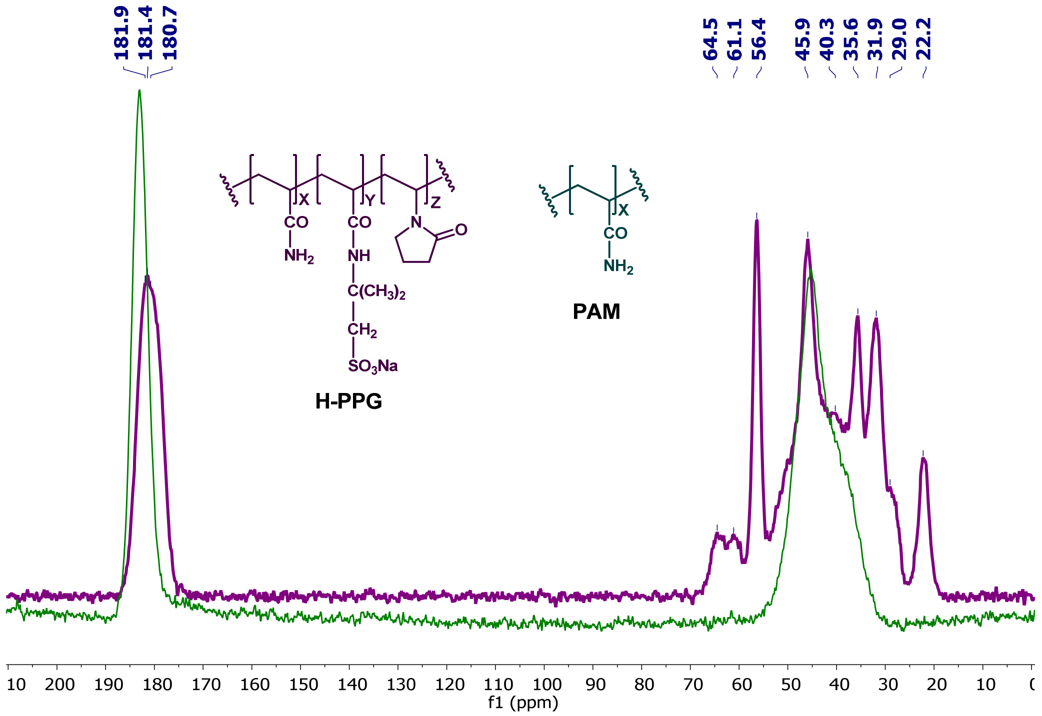

Figure S5. Overlapping spectra of ^{13}C NMR CP/MAS: terpolymer **H-PPG** (line purple) and homopolymer **polyacrylamide** (line green), acquired to 150 MHz.

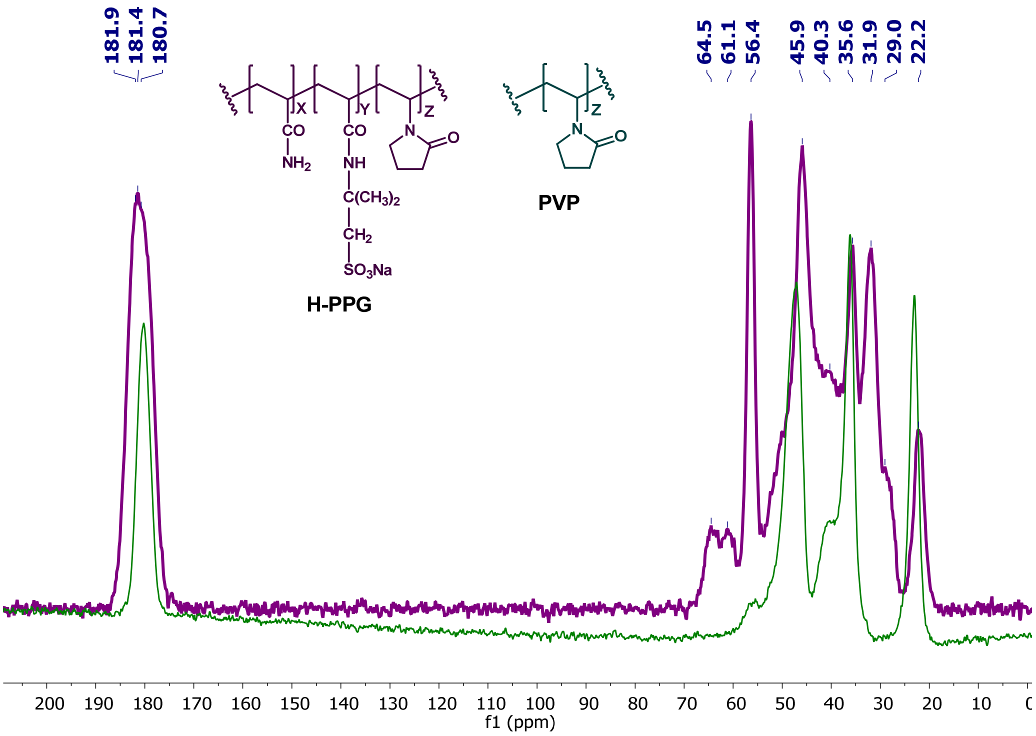

Figure S6. Overlapping spectra of ^{13}C NMR CP/MAS: terpolymer **H-PPG** (line purple) and homopolymer **polyvinylpyrrolidone** (line green), acquired to 150 MHz.

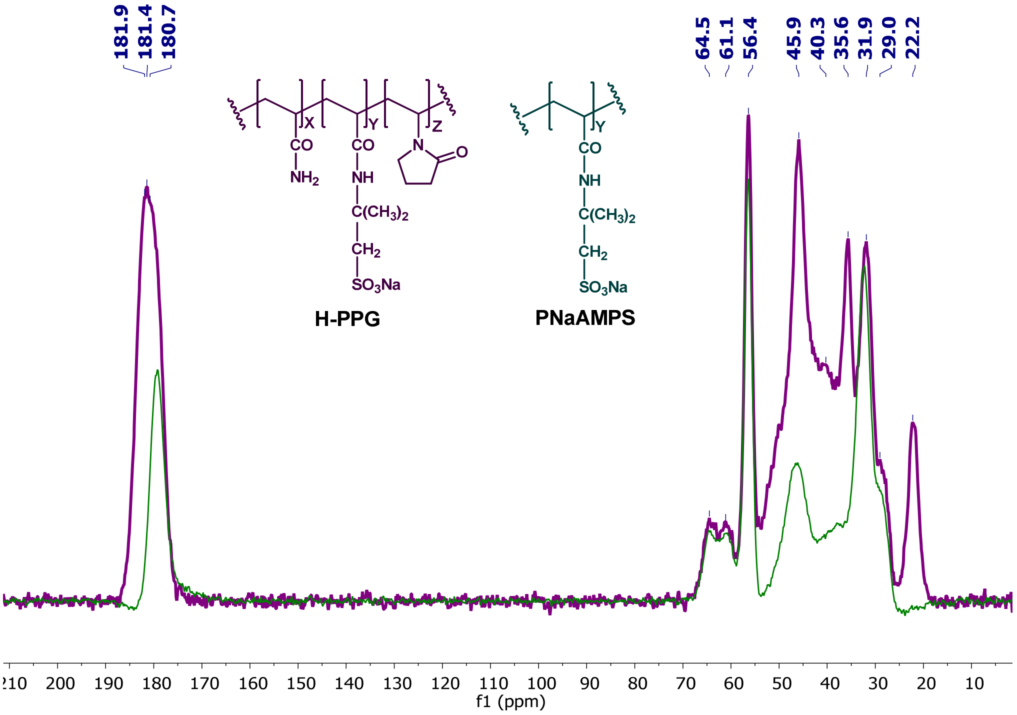

Figure S7. Overlapping spectra of ^{13}C NMR CP/MAS: terpolymer **H-PPG** (line purple) and homopolymer **polyNaAMPS** (line green), acquired at 150 MHz.

Table S4. Assignment of the signals of the spectra ^{13}C NMR of the polymers: **H-PPG**, **PAM,** **PVP**, and **PNaAMPS**.

Signal type	Chemical shift (ppm)			
	H-PPG	PAM	PVP	PNaAMPS
-C=O	181.9	183.0	-	-
-C=O	181.4	-	180.2	-
-C=O	180.7	-	-	179.5
	64.5	-	-	64.6
$-CH_2-$	61.1	-	-	61.1
-C-	56.4	-	55.5	56.4
$-CH_2-$	45.9	43.3	47.0	46.1
$-CH_2-$	40.3	-	40.3	-
$-CH_2-$	35.6	-	36.1	-
$-CH_3-$	31.9	-	-	32.3
-CH-	29.0	-	-	28.9
$-CH_2-$	22.2	-	23.0	-

Compound: P-PPG

Figures **S8-S10** show the overlapping ^{13}C NMR CP/MAS spectra of the terpolymer P-PPG (purple line) with polyacrylamide, polyvinylpyrrolidone, and polyNaAMPS (green line), respectively. The signs generated by each homopolymer coincide with the chemical shift of the signs present in the spectrum of terpolymer **P-PPG**. **Table S5** describes the signals present in the overlapping spectra.

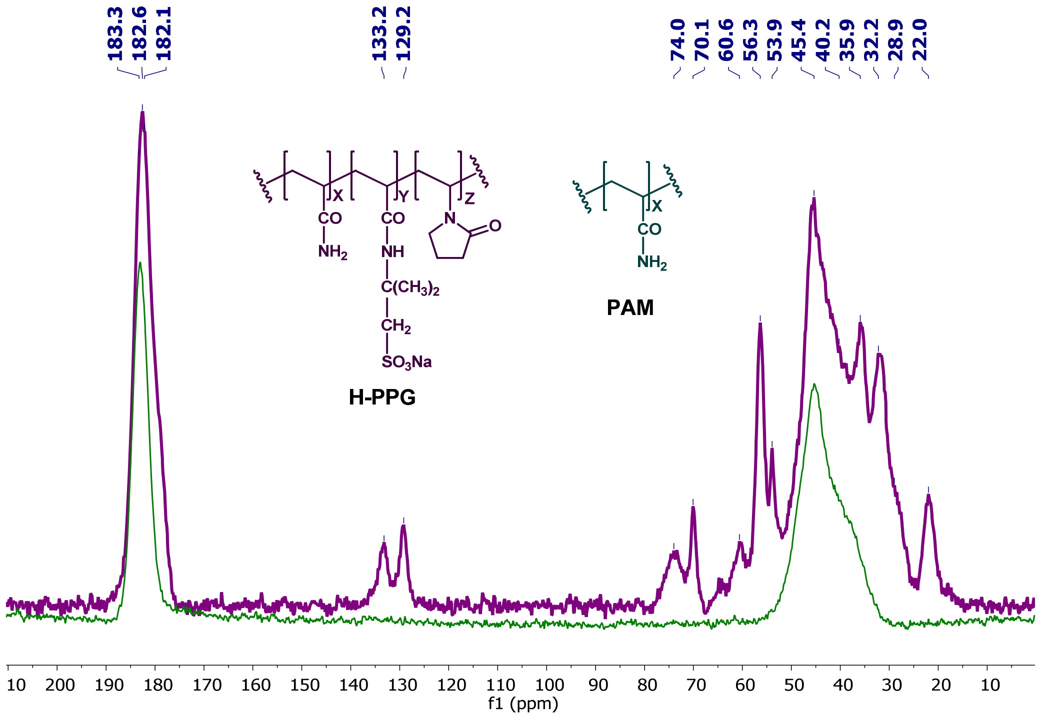

Figure S8. Overlapping spectra of ^{13}C NMR CP/MAS: terpolymer **P-PPG** (line purple) and homopolymer **polyacrylamide** (line green), acquired at 150 MHz.

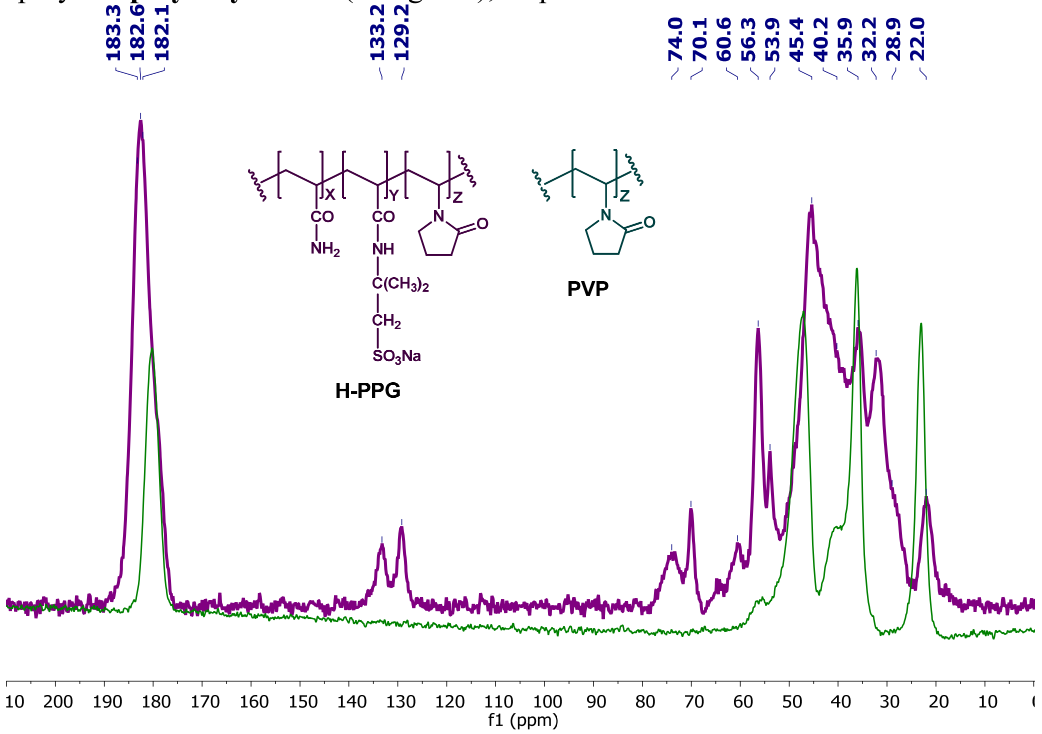

Figure S9. Overlapping spectra of ^{13}C NMR CP/MAS: terpolymer **P-PPG** (line purple) and homopolymer **polyvinylpyrrolidone** (line green), acquired at 150 MHz.

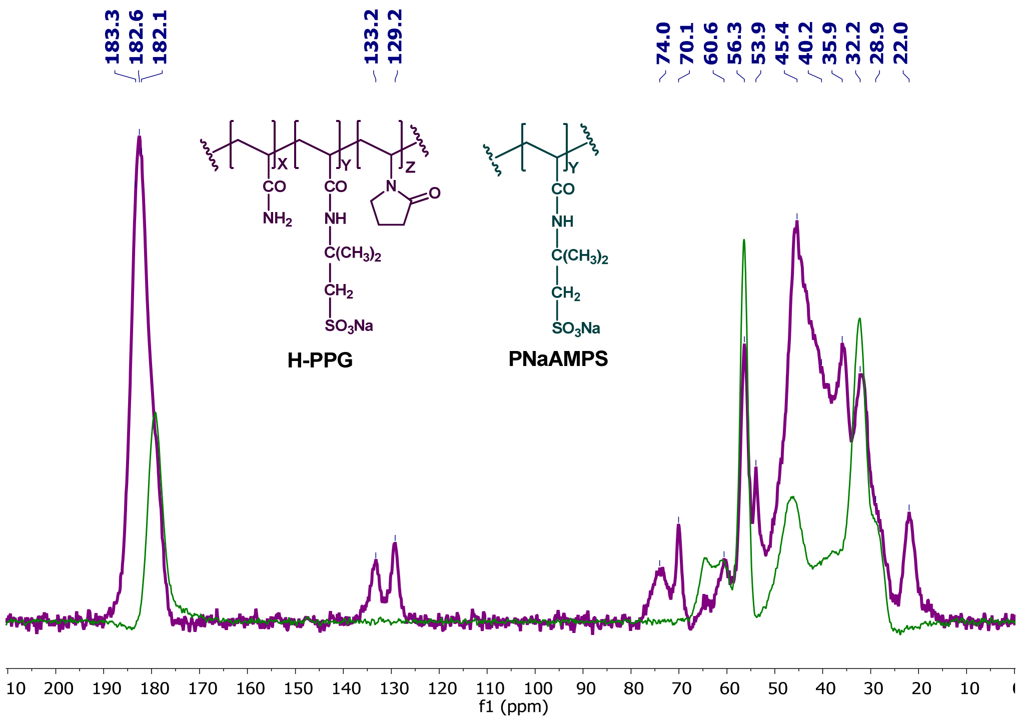

Figure S10. Overlapping spectra of ^{13}C NMR CP/MAS: terpolymer **P-PPG** (line purple) and homopolymer **polyNaAMPS** (line green), acquired at 150 MHz.

Table S5. Assignation of the signals of the spectra ^{13}C NMR of the polymers: **P-PPG**, **PAM, PVP**, and **PNaAMPS**.

**Signal type**	**Chemical shift (ppm)**					
	**H-PPG**	**PAM**	**PVP**	**PNaAMPS**	**DADMAC[1]**	**PDADMAC[2]**
-C=O	183.3				-	
-C=O	182.6	183.0	180.2	179.5	-	
-C=O	180.1				-	
CH_2=	133.2	-	-	-	129.1	
=CH-	129.2	-	-	-	124.4	
$-CH_2-$	74.1	-	-	-	-	71.8
$-CH_2-$	70.1	-	-	-	-	71.1
$-CH_2-$	60.6	-	-	61.1	66.1	
-C-	56.3	-	55.5	56.4	-	
CH_3-	53.9	-	-	-	53.9	56.9
$-CH_2-$	45.4	43.3	47.0	46.1	-	39.0, 44.0
$-CH_2-$	40.2	-	40.3	-	-	
$-CH_2-$	35.9	-	36.1	-	-	
$-CH_3-$	32.2	-	-	32.3	-	
-CH-	28.9	-	-	28.9	-	
$-CH_2-$	22.0	-	23.0	-	-	
DADMAC: Diallyldimethylammonium chloride.<br>PDADMAC: Poly(diallyldimethylammonium chloride).						

[1] Zhang Y. J.; Jia X. Synthesis of Ultra High Molecular Weight Poly(dimethyldiallyl ammonium chloride). *Russ. J. Appl. Chem.* **2016**, 89, 315-323.

[2] Brand F.; Dautzenberg H.; Jaeger W.; Hahn M. Polyelectrolytes with various charge densities: Synthesis and characterization of diallyldimethylammonium chloride-acrylamide copolymers. *Angew. Makromolek. Chem.* **1997**, 248, 41-71.

Spectroscopic characterization of terpolymers by FTIR-ATR.

Compound: H-PPG

In the same way, FTIR-ATR spectra were compared. Figures **S11-S13** show the FTIR-ATR spectra of the terpolymer H-PPG (purple line) overlapped with those of polyacrylamide, polyvinylpyrrolidone, and polyNaAMPS (green line), respectively. The bands generated by each homopolymer coincide with those present in the spectrum of the terpolymer **H-PPG**. **Table S6** describes the bands present in the overlapping spectra.

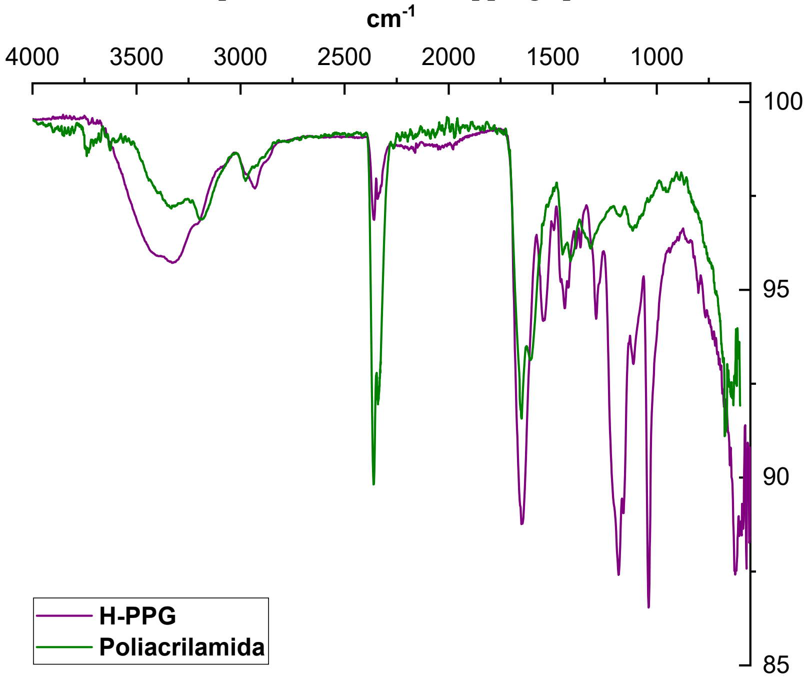

Figure S11. Overlapping spectra of FTIR-ATR: terpolymer **H-PPG** (line purple) and homopolymer **polyacrylamide** (line green).

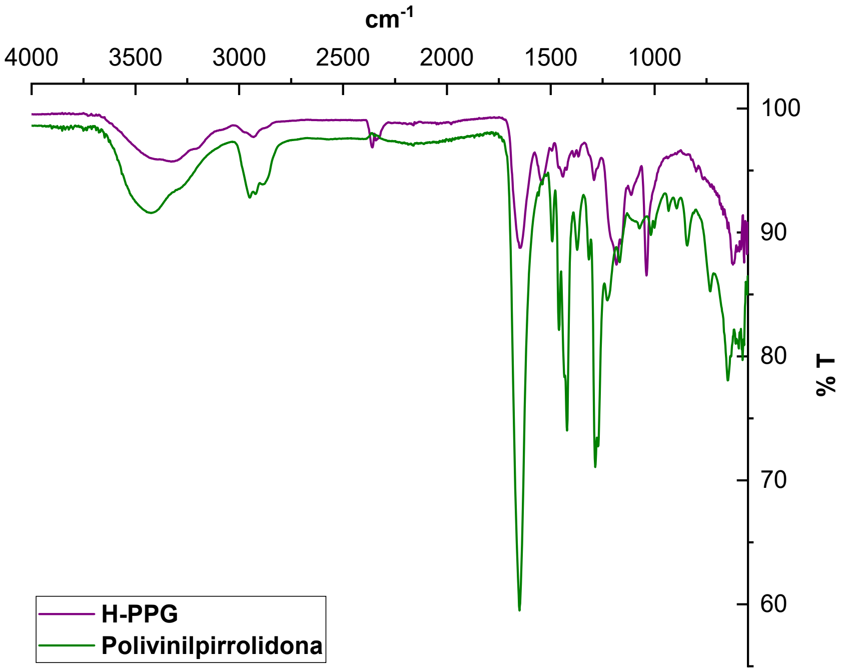

Figure S12. Overlapping spectra of FTIR-ATR: terpolymer **H-PPG** (line purple) and homopolymer **polyvinylpyrrolidone** (line green).

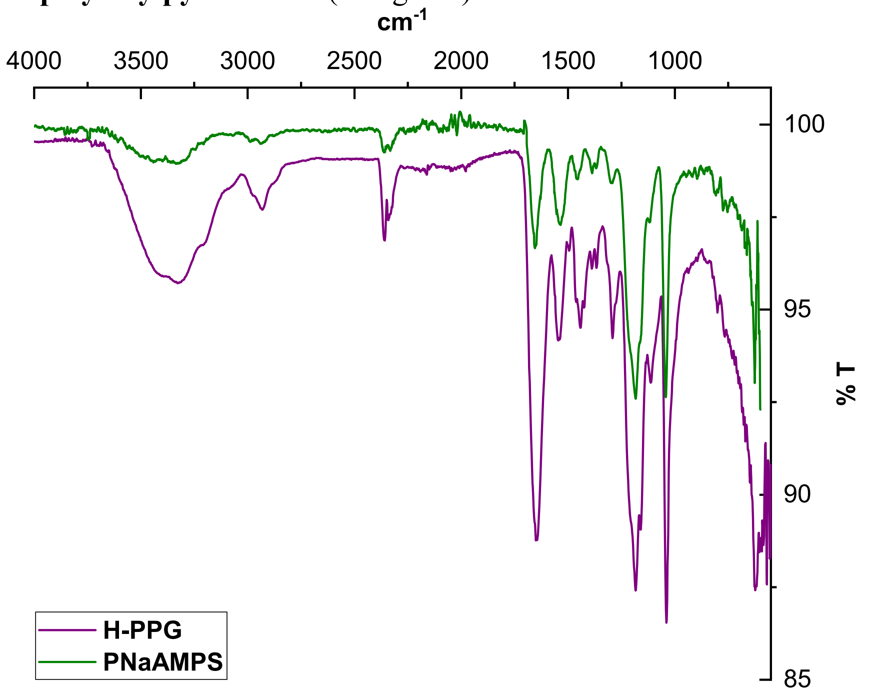

Figure S13. Overlapping spectra of FTIR-ATR: terpolymer **H-PPG** (line purple) and homopolymer **polyNaAMPS** (line green).

Table S6. Associated bands to the functional groups present in the polymers **H-PPG**, **PAM**, **PVP,** and **PNaAMPS**.

Functional groups	Wave number υ (cm^{-1})[3]			
	**H-PPG**	**PAM**	**PVP**	**PNaAMPS**
OH, and/or -NH/-NH$_2$	3405, 3325	3334, 3189	3308	3439, 3325, 1538
-CH$_2$ and/or -CH$_3$	2976, 2936	2977, 1455, 1316	2984, 2887, 1284	2989, 2937
-CO-N-(R)$_2$	1652	1650	1664	1653
R-N-(H$_2$)R$_2$			1214, 1094	
-C(CH$_3$)$_2$-	1184	-	-	1184
-S=O	1039	-	-	1041

Compound: P-PPG

Figures **S14-S16** show the FTIR-ATR spectra of the terpolymer P-PPG (purple line) overlapped with those of polyacrylamide, polyvinylpyrrolidone, and polyNaAMPS (green line), respectively. The bands generated by each homopolymer coincide with those present in the spectrum of the terpolymer **P-PPG**. **Table S7** describes the bands present in the overlapping spectra.

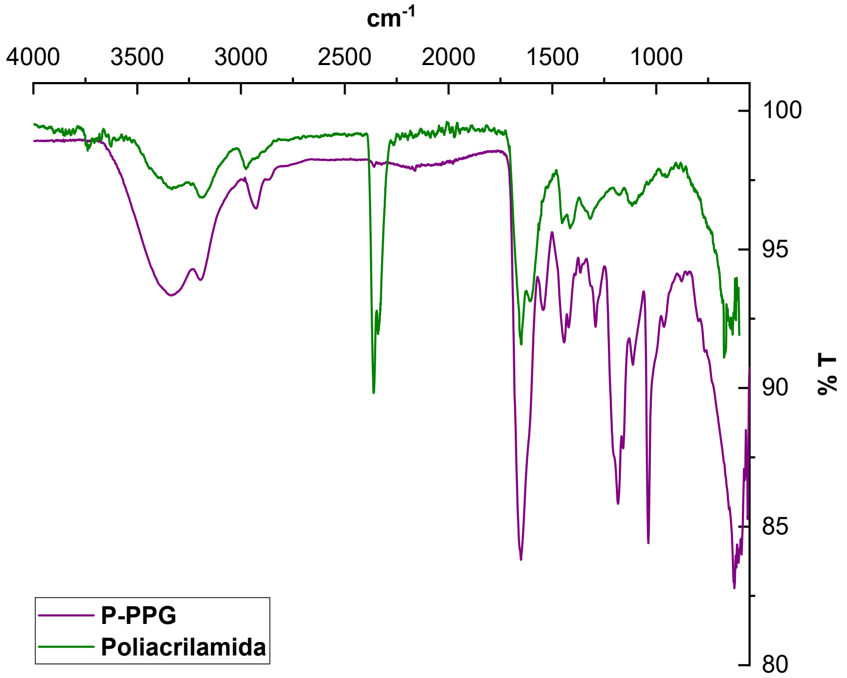

Figure S14. Overlapping spectra of FTIR-ATR: terpolymer **P-PPG** (line purple) and homopolymer **polyacrylamide** (line green).

[3] Nakanishi K. Infrared Absorption Spectroscopy Practical; Nankodo Company Limited: Tokyo, Japan, 1969.

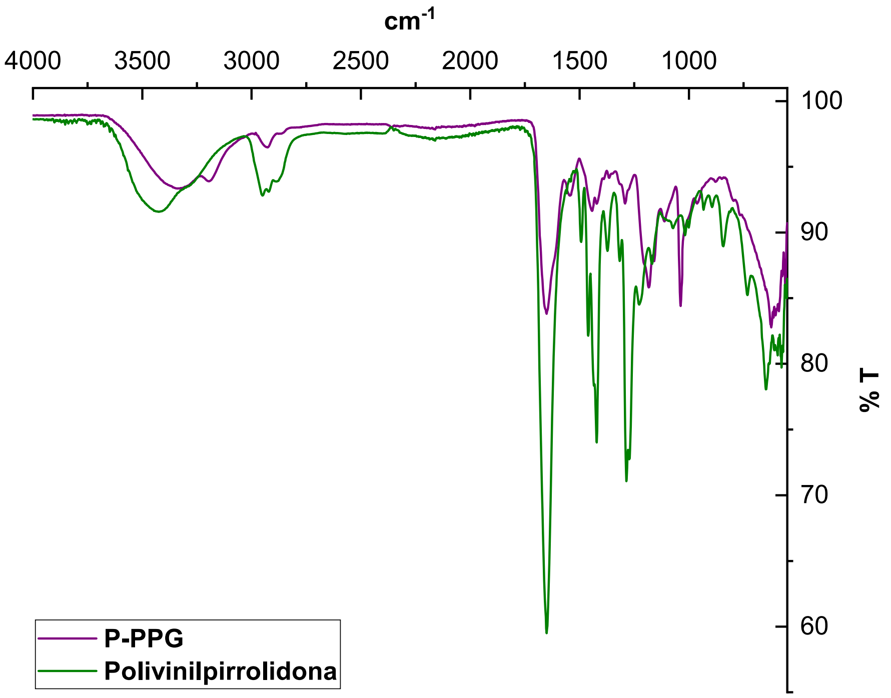

Figure S15. Overlapping spectra of FTIR-ATR: terpolymer **P-PPG** (line purple) and homopolymer **polyvinylpyrrolidone** (line green).

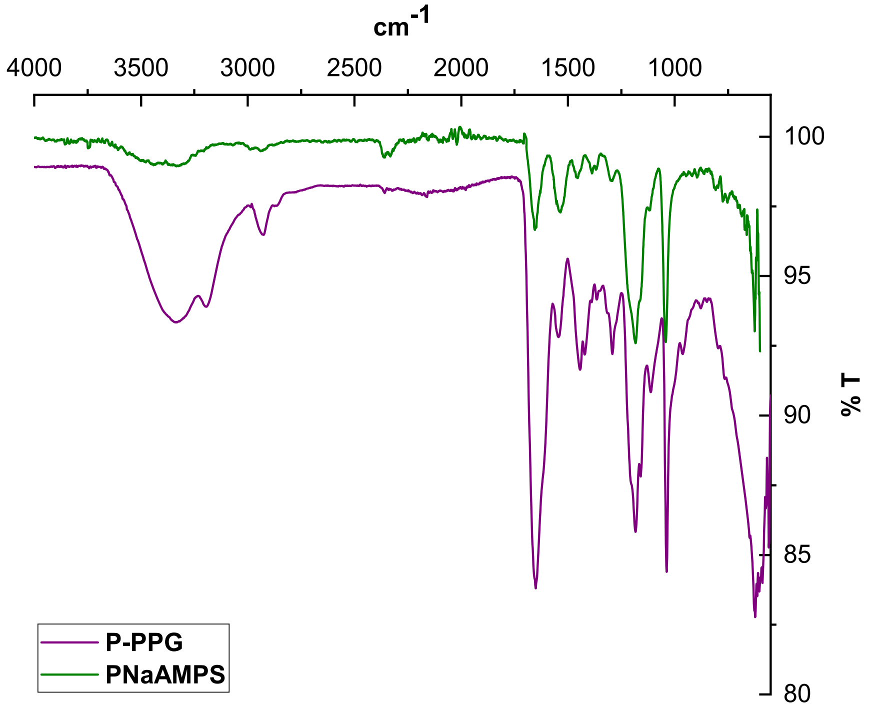

Figure S16. Overlapping spectra of FTIR-ATR: terpolymer **P-PPG** (line purple) and homopolymer **polyNaAMPS** (line green).

Table S7. Associated bands to the functional groups present in the polymers **P-PPG**, **PAM**, **PVP** and **PNaAMPS**.

Functional groups	Wave number υ (cm^{-1})[3]			
	H-PPG	PAM	PVP	PNaAMPS
OH, and/or -NH/-NH_2	3405, 3325	3334, 3189	3308	3439, 3325, 1538
-CH_2 and/or -CH_3	2976, 2936	2977, 1455, 1316	2984, 2887, 1284	2989, 2937
-CO-N-$(R)_2$	1652	1650	1664	1653
R-N-$(H_2)R_2$			1214, 1094	
-$C(CH_3)_2$-	1184	-	-	1184
-S=O	1039	-	-	1041

Characterization of terpolymers by Elemental Analysis.

Compound: **H-PPG** and **P-PPG**

The non-normalized results of the elemental analysis of samples **H-PPG** and **P-PPG** are shown in **Table S8**.

Table S8. Results of elemental analysis of terpolymers **H-PPG** and **P-PPG.**

Sample	% Nitrogen	% Carbon	% Hydrogen	% Sulfur	% Oxygen
**H-PPG**	8.6	37.5	6.0	6.4	27.3
**P-PPG**	10.6	40.8	6.9	3.3	23.0

For the effects of calculating the empirical formula and considering only the elements determined in the present, the values found were normalized from the elemental analysis carried out. **Table S9** shows the normalized data.

Table S9. Elemental analysis percentages of normalized **H-PPG** and **P-PPG** Samples.

Sample	% Nitrogen	% Carbon	% Hydrogen	% Sulfur	% Oxygen
**H-PPG**	10.0	43.7	7.0	7.5	31.8
**P-PPG**	12.5	48.2	8.2	3.9	27.2

Using the values from Table S9, the empirical formulas for the chemical products H-PPG and P-PPG were calculated. The total molecular weight as a fundamental unit was 855.093 g/mol for H-PPG and 1644.41g/mol for P-PPG. Finally, the highest probability of empirical formulas is listed below, **Table S10**:

Table S10. Empirical formulas of samples of terpolymers **H-PPG** and **P-PPG**.

Terpolymer	Empirical formula
**H-PPG**	C_{31}, H_{59}, N_6, S_2, O_{17}
**P-PPG**	C_{66}, H_{134}, N_{15}, S_2, O_{28}

The uncorrected elemental analysis data (not normalized) indicate that a percentage of 14.2 in **H-PPG** and 15.4 % in **P-PPG** do not correspond to the elements carbon, hydrogen,

nitrogen, sulfur, or oxygen of an organic nature, for which presumptively said percentages correspond to the components of bentonite, which is composed of aluminum silicates and other elements. The empirical formulas reveal that the polymers contain sulfur, confirming the incorporation of NaAMPS into the terpolymers. It can also be appreciated that oxygen and nitrogen molecules are constituents of the theoretical molecules.

Characterization of terpolymers by TGA/DSC.

Thermal analysis of hydrogels was conducted using a TGA/DSC 1 and an HP DSC 1 (Mettler-Toledo Inc., Switzerland) within the temperature range of 25–500 ºC at a heating rate of 10 °C/min under nitrogen.

Figures S17 and S18 display the TGA/DSC profiles of P-PPG and its individual homopolymers. P-PPG has a glass transition temperature of 62°C (Figure S17)and begins to decompose at 323°C. The data show that its thermal properties are mainly affected by the AMPSNa homopolymer. There are two stages of decomposition (Figure S18). The first stage, occurring between 50 ºC and 100 °C and showing slight weight loss, is caused by the evaporation of physically trapped moisture. The second stage captures the H-PPG decomposition that starts at 323°C.

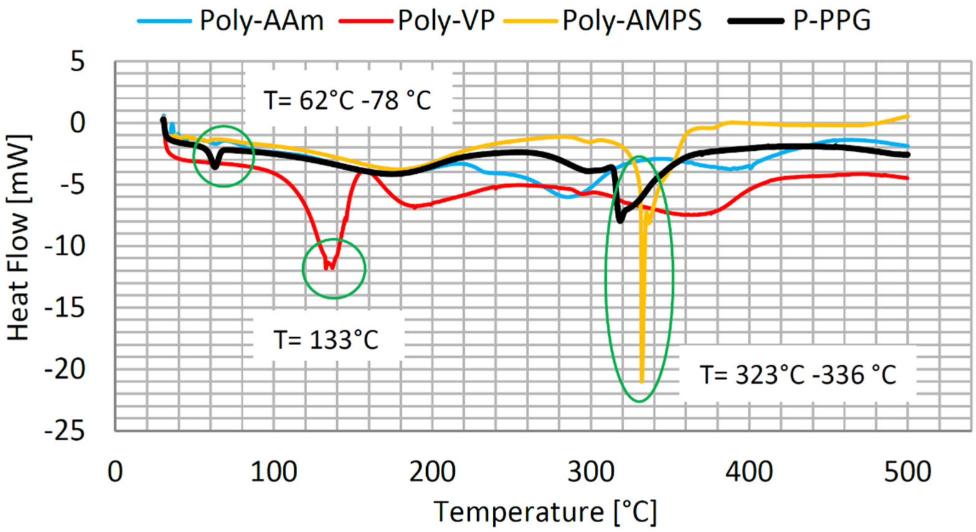

Figure S17. DSC curves of the terpolymer **Poly-AAm**, **Poly-VP**, **Poly-PAMPS,** and **P-PPG**.

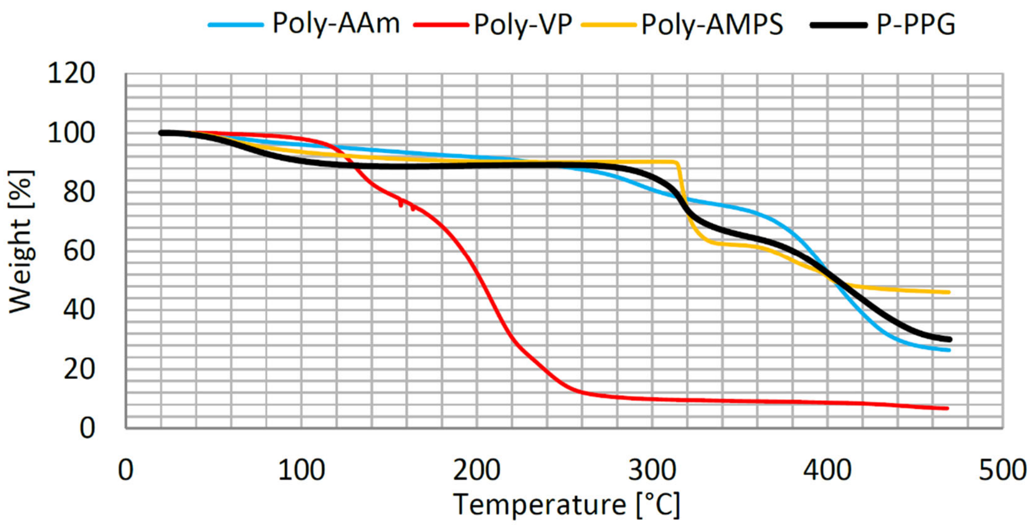

Figure S18. Comparison of TGA curves of the terpolymer **Poly-AAm**, **Poly-VP**, **Poly-PAMPS,** and **P-PPG**.

Figures S19 and S20 display the TGA/DSC profiles of P-PPG and its individual homopolymers. P-PPG has a glass transition temperature of 62°C (Figure S19) and begins to decompose at 323°C. The data show that its thermal properties are mainly affected by the AMPSNa homopolymer. There are two stages of decomposition (Figure S20). The first stage, occurring between 50 ºC and 100 °C and showing slight weight loss, is caused by the evaporation of physically trapped moisture. The second stage captures the H-PPG decomposition that starts at 323°C.

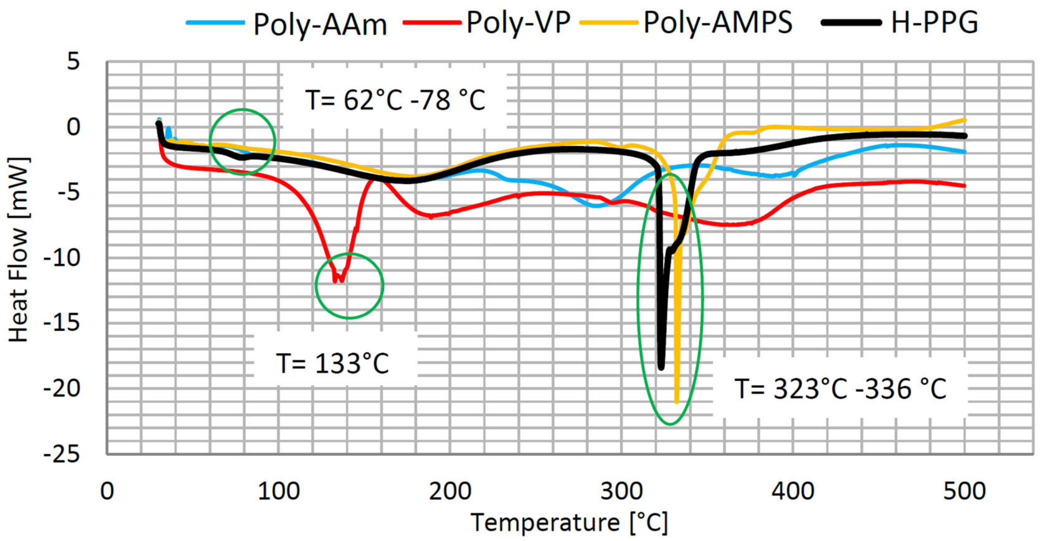

Figure S19. DSC curves of the terpolymer **Poly-AAm**, **Poly-VP**, **Poly-PAMPS,** and **H-PPG**.

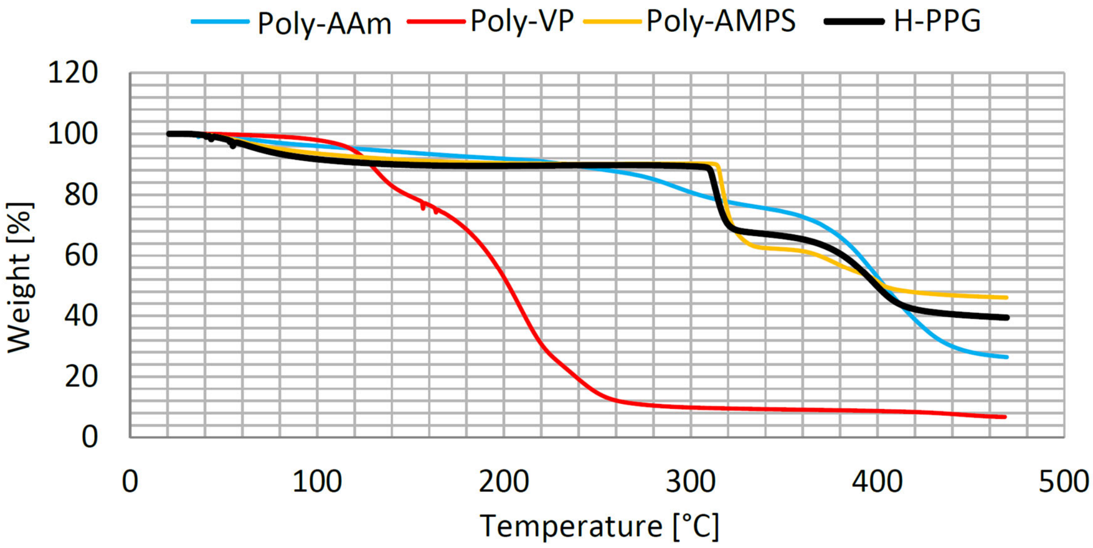

Figure S20. TGA curves of the terpolymer **Poly-AAm**, **Poly-VP**, **Poly-PAMPS,** and **H-PPG**.

Characterization of terpolymers by Environmental Scanning Electron Microscope (ESEM).

Figures S21 and S22 display environmental scanning electron microscopy (ESEM) images of the H-PPG and P-PPG terpolymers. The samples were first allowed to swell for 24 hours in deionized water (Figure S21) or in formation brine (Figure S22), then lyophilized.

Scale	P-PPG	H-PPG
10 µm	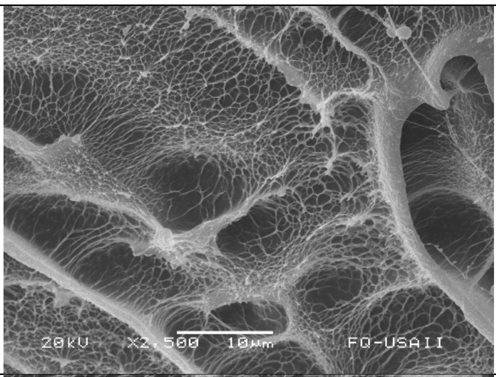	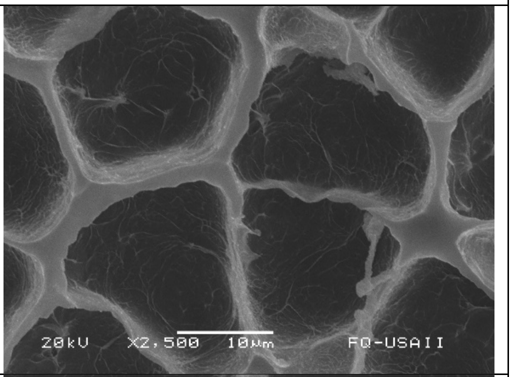
5 µm	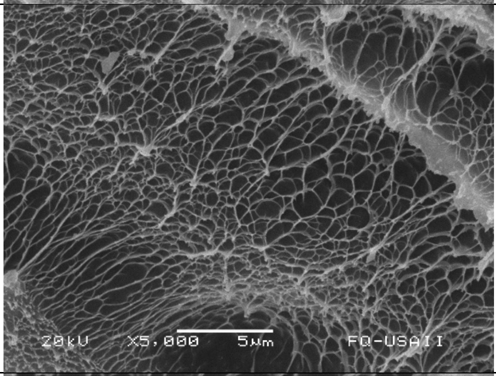	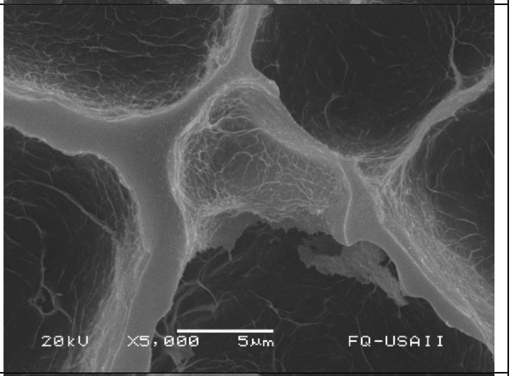
1 µm	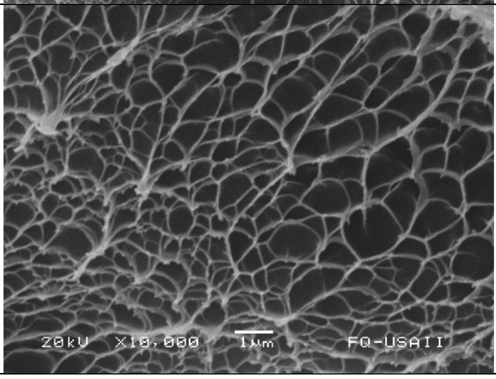	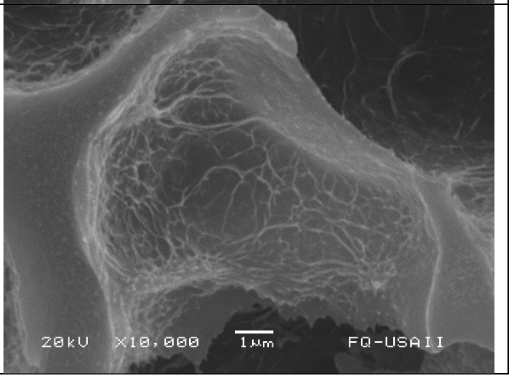

Figure S21. ESEM micrographs of the **P-PPG** and **H-PPG** terpolymers reveal pore structures resulting from swelling in desionized water.

Scale	P-PPG	H-PPG
10 µm	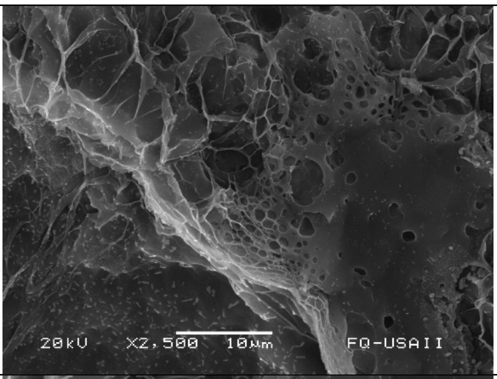	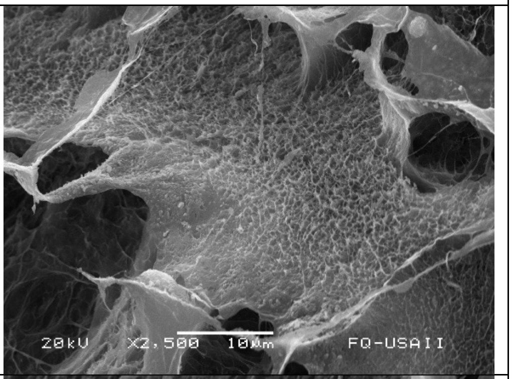
5 µm	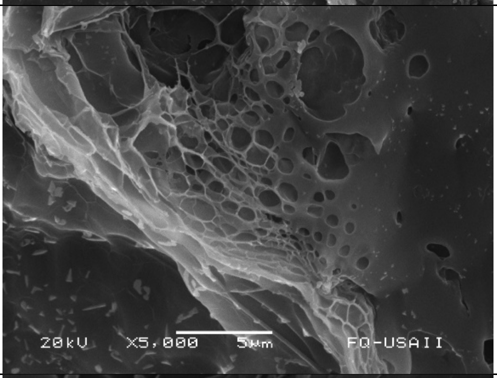	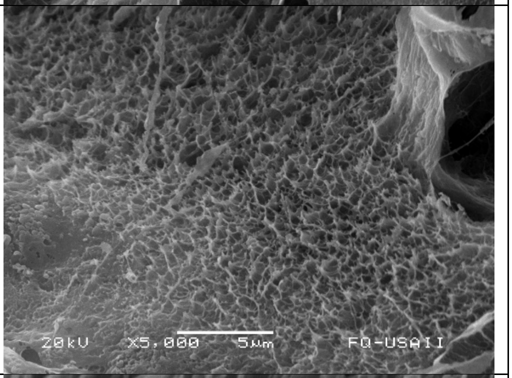
1 µm	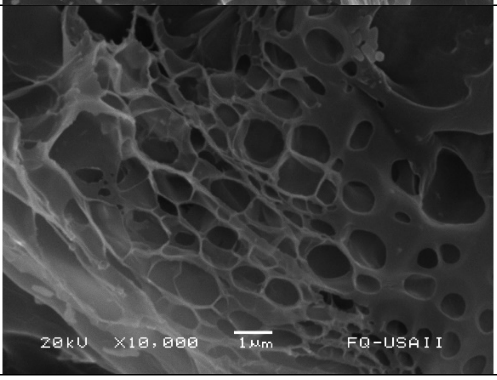	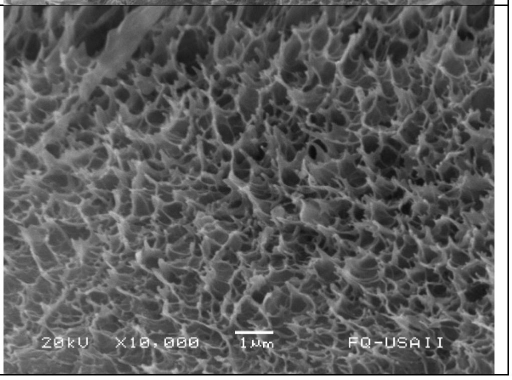

Figure S22. ESEM micrographs of the **P-PPG** and **H-PPG** terpolymers reveal pore structures resulting from swelling in formation brine.

SEM images reveal the morphologies of the pore structures of P-PPG and H-PPG. In deionized water (Figure S21), P-PPG shows a narrow, uniform pore-size distribution, ensuring consistent performance, reducing stress concentrations, and preventing premature failures. H-PPG's network is less dense, with non-dispersed HPAAm present, resulting in non-swollen, compacted boundaries. Swelling in formation brine (Figure S22) makes the dual network of H-PPG pores more uniform and denser than that of P-PPG.

Table S11. Elemental compositions of the terpolymers **H-PPG** and **P-PPG**.
Thermal JEOL.

**Terpolymers**	**Concentration [% w]**							
	**Carbon**	**Oxygen**	**Nitrogen**	**Sulfur**	**Sodium**	**Silicon**	**Aluminum**	**Chlorine**
**H-PPG**	50.3	27.8	10.6	5	4.3	1	0.9	0
**P-PPG**	51.1	10.7	7.8	1.2	13.7	0.4	0.2	14.9

Optimization of components of P-PPG and H-PPG.

1). Optimization:

a. Overall composition: 30% wt.

b. Gel type behavior under thermal stability at 130°C for 90 days, based on the Sydansk gel-strength code[4].

c. Evolution of the viscoelastic behavior of aged samples at 130°C for 90 days.

Components: Acrylamide (AAm), Vinylpyrrolidone (VP), Sodium 2-acrylamido-2-methylpropane sulfonic (AMPSNa), Diallyldimethylammonium Chloride (DADMAC), N,N'-Methylenebis(acrylamide) (MBA), 40 % Hydrolyzed Polyacrylamide (HPAAm), Modified Bentonite (MB), Ammonium persulfate (APS), N,N,N′,N′-Tetramethyl ethylenediamine (TEMED)

Table S12. Compositional range for **P-PPG** optimization.

Conc. Limit	Total Conc.	AAm	VP	AMPSNa	DADMAC	MBA	MB	APS	TEMED
	% wt	Molar ratio				% wt.			
low	10	1	0.5	0.5	0.5	0.25	0.5	0.05	0.05
High	30	2.5	1	1.5	1.5	0.75	2	0.5	0.5

Table S13. Compositional range for **H-PPG** optimization.

Conc. Limit	Total Conc.	AAm	VP	AMPSNa	HPAAm	MBA	MB	APS
	% wt	Molar ratio			% wt.			
low	10	1	0.5	0.5	0.5	0.25	0	0.05
High	30	2.5	1	1.5	2	0.75	2	0.5

[4] Sydansk, R.D. A new Conformance-Improvement-Treatment Chromium(III) Gel technology. In Proceedings of the SPE Enhanced Oil Recovery Symposium, Society of Petroleum Engineers, Tulsa, Oklahoma, 16–21 April 1988; pp. 99–111. https://doi.org/10.2118/17329-MS.

Evolution of the linear viscoelastic region of PPGs, during aging with formation brine at 130 °C

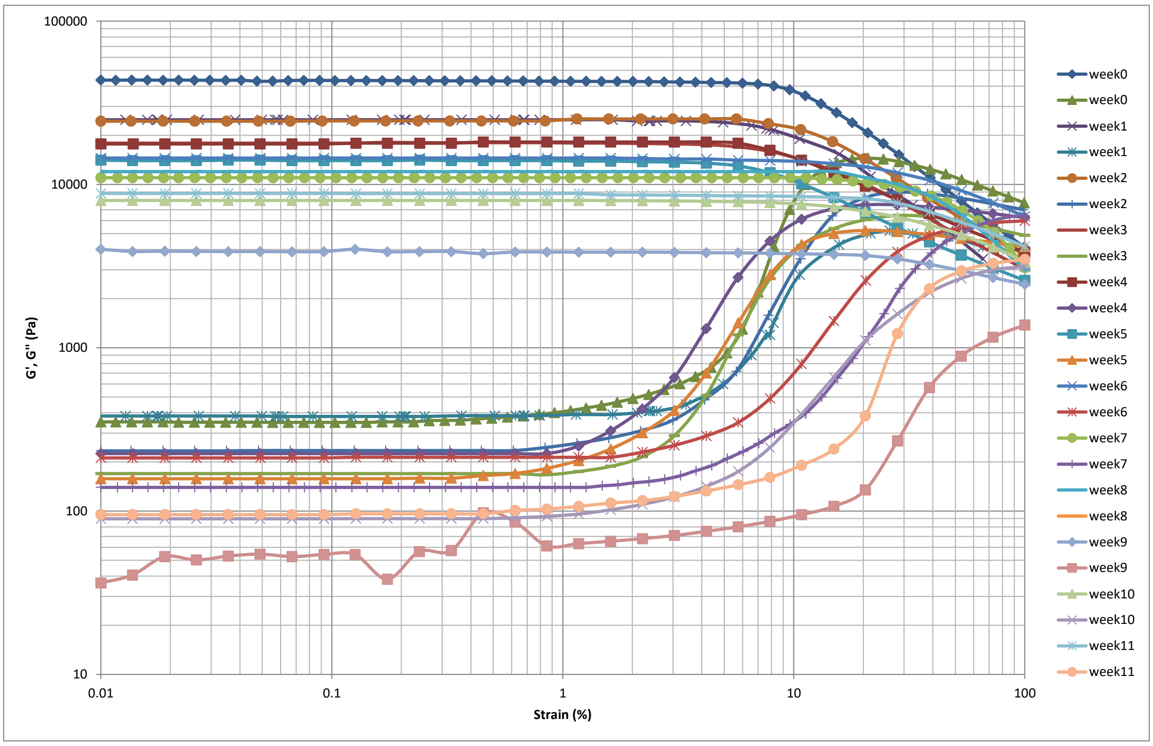

Figure S23. Evolution of the linear viscoelastic region of C-PPG, during aging with formation brine at 130 °C.

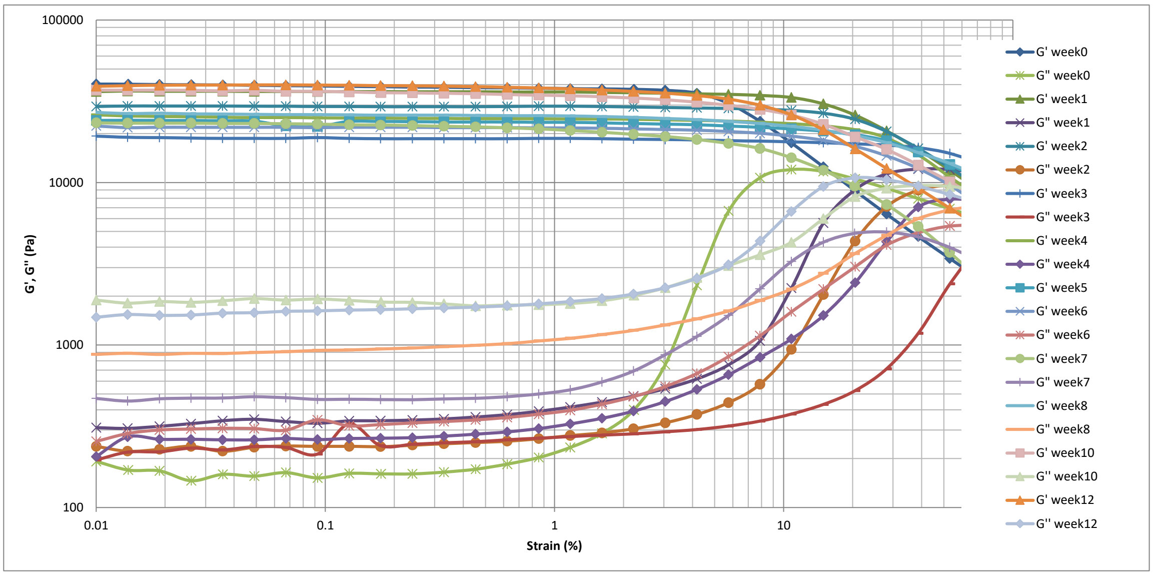

Figure S24. Evolution of the linear viscoelastic region of P-PPG, during aging with formation brine at 130 °C.

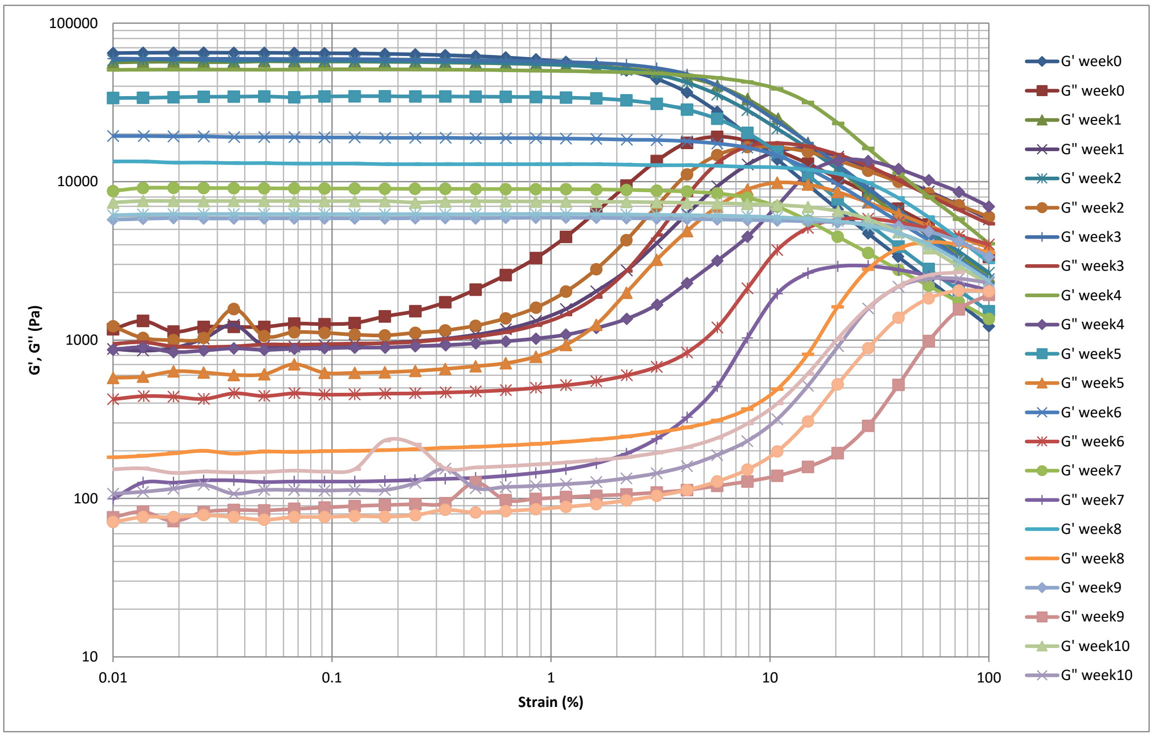

Figure S25. Evolution of the linear viscoelastic region of H-PPG, during aging with formation brine at 130 °C.
